# OMMA enables population-scale analysis of complex genomic features and phylogenomic relationships from nanochannel-based optical maps

**DOI:** 10.1093/gigascience/giz079

**Published:** 2019-07-09

**Authors:** Alden King-Yung Leung, Melissa Chun-Jiao Liu, Le Li, Yvonne Yuk-Yin Lai, Catherine Chu, Pui-Yan Kwok, Pak-Leung Ho, Kevin Y Yip, Ting-Fung Chan

**Affiliations:** 1 School of Life Sciences, The Chinese University of Hong Kong, Shatin, Hong Kong; 2 Carol Yu Center for Infection and Department of Microbiology, The University of Hong Kong, Queen Mary Hospital, Pok Fu Lam, Hong Kong; 3 Department of Computer Science and Engineering, The Chinese University of Hong Kong, Shatin, Hong Kong; 4 Cardiovascular Research Institute, University of California, San Francisco, CA 94153, USA; 5 Institute of Human Genetics, University of California, San Francisco, CA 94153, USA; 6 State Key Laboratory of Agrobiotechnology, The Chinese University of Hong Kong, Shatin, Hong Kong; 7 Hong Kong Bioinformatics Centre, The Chinese University of Hong Kong, Shatin, Hong Kong

**Keywords:** optical mapping, comparative genomics, structural variation, copy number variation, haplotypes, single-molecule analysis

## Abstract

**Background:**

Optical mapping is an emerging technology that complements sequencing-based methods in genome analysis. It is widely used in improving genome assemblies and detecting structural variations by providing information over much longer (up to 1 Mb) reads. Current standards in optical mapping analysis involve assembling optical maps into contigs and aligning them to a reference, which is limited to pairwise comparison and becomes bias-prone when analyzing multiple samples.

**Findings:**

We present a new method, OMMA, that extends optical mapping to the study of complex genomic features by simultaneously interrogating optical maps across many samples in a reference-independent manner. OMMA captures and characterizes complex genomic features, e.g., multiple haplotypes, copy number variations, and subtelomeric structures when applied to 154 human samples across the 26 populations sequenced in the 1000 Genomes Project. For small genomes such as pathogenic bacteria, OMMA accurately reconstructs the phylogenomic relationships and identifies functional elements across 21 *Acinetobacter baumannii* strains.

**Conclusions:**

With the increasing data throughput of optical mapping system, the use of this technology in comparative genome analysis across many samples will become feasible. OMMA is a timely solution that can address such computational need. The OMMA software is available at https://github.com/TF-Chan-Lab/OMTools.

## Findings

### Background

Optical mapping captures the labeling patterns of long DNA molecules and has become a complementary approach to sequencing-based methods [1]. DNA labels are usually created by a short but specific nicking restriction enzyme (nickase), but alternative labeling strategies, such as methylations [2, 3] and sequence-specific labeling based on CRISPR technology [4] have also been described. With optical map ranges from 100 kb to as high as 1 Mb, optical mapping is well suited to assist with sequence scaffolding in genome assembly and in the detection of large structural variations.

Multiple alignment (MA) is a process in which multiple queries are aligned without relying upon a reference. This type of comparison is especially useful when a standard reference is not available or is of poor quality. Also, in certain variable regions, the alignment of multiple queries to a reference could only show a 1-to-1 difference between the individual query and the reference. In contrast, MA of these queries helps to differentiate groups of patterns among the queries. Unlike pairwise alignment, for which many algorithms have been designed [5–10], MA remains an underdeveloped method for optical mapping. The other available tool is included in the proprietary software Bionumerics v7 [11], but this method is not sensitive to genomic rearrangement and requires complete genomes as inputs.

We developed a novel MA algorithm for population-scale analysis of optical mapping data: optical mapping by multiple alignment (OMMA). The OMMA program was designed for comparing assembled optical maps, which are usually longer than raw optical maps, and with the intrinsic errors that have already been addressed during the *de novo* assembly steps. We describe herein the algorithm and demonstrate its effectiveness with both simulated and experimental data. We also demonstrate how OMMA can be used to resolve complex genomic features and reconstruct phylogenomic relationships.

### OMMA pipeline

**Figure 1:**
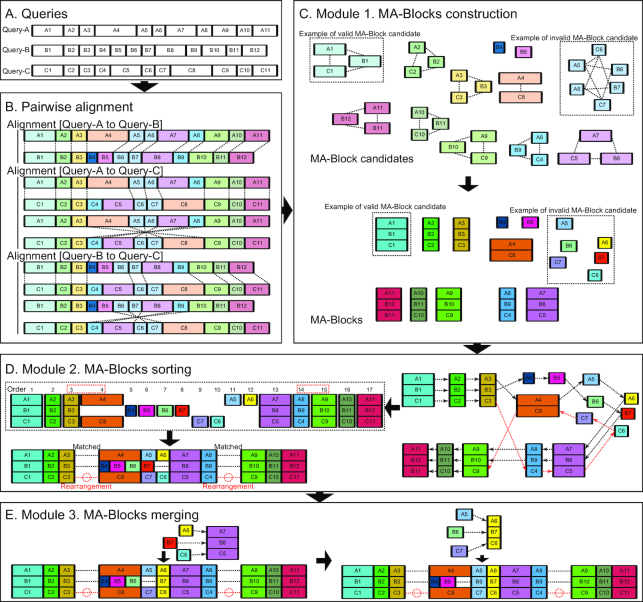
Overview of the OMMA pipeline. Details of these algorithms are described in Methods. (A) Example queries for multiple alignment. (B) Pairwise alignments of the queries to create sources for segment links. An insertion/deletion event occurs between queries A and B (see segments A4, B4, and B5). An inversion event occurs between queries A and C (see segments A4–A8 and C4–C8). (C) MA-block construction. An undirected graph is constructed with segments as vertices and segment links as edges. An MA-block candidate (connected component in the undirected graph) that fulfills the MA-block criteria (e.g., [A1, B1, C1]) is converted directly into an MA-block [A1, B1, C1]. In contrast, segments in an MA-block candidate that does not fulfill the criteria (e.g., [A5, A6, B6, B7, C6, C7]) are broken into individual MA-blocks [A5], [A6], [B6], [B7], [C6], and [C7]. (D) MA-block sorting. Matching events between 2 MA-blocks (dotted arrow) are determined by any proximate segment (e.g., A1 to A2 or C3 to C4). Red dotted arrows highlight matching events within the inverted region of query C. Their suggested direction is opposite that from queries A and B. The MA-blocks are sorted to minimize rearrangement events. Here 2 rearrangement events occur when joining segments C3–C8 (MA-blocks 3 and 4) and C4–C9 (MA-blocks 14 and 15). The sorted MA-blocks are packed for purposes of visualization, with black dotted lines indicating empty space, and red dotted circles indicating rearrangement events. (E) MA-block merging. Without contradicting the order of MA-blocks determined in the last module, MA-blocks that share similar segment sizes are merged. MA-blocks [A6], [B7], and [C6] share a similar size and are proximate to the MA-block [A7, B8, C5] (purple) and are merged into 1 MA-block. Similarly, MA-blocks [A5], [B6], and [C7] share a similar size and are proximate to the MA-block [A6, B7, C6] (yellow) and are merged into another MA-block.

The complete OMMA pipeline has 2 steps—the preparation step and the MA step (Fig. 1). During the preparation step, optical maps (Fig. 1A) are aligned in a pairwise manner (Fig. 1B). Two segments from 2 different optical maps are said to match if their left and right labels are both aligned. In the MA step, information about the matching segments is used to produce chains of MA-blocks as the MA results; an MA-block is defined as a collection of segments, each from a different optical map, that all match each other. This step can be further divided into 3 main substeps (modules) (Fig. 1C–E). In the first substep, MA-blocks are formed on the basis of the results of the preparation step (Fig. 1C). In the second substep, the MA-blocks are sorted to maximize matching and to minimize rearrangement events (Fig. 1D). Finally, in the third substep, proximate MA-blocks that are similar to each other are merged (Fig. 1E). The details of the pipeline are described in the Methods.

### Performance analysis

In this section we describe the accuracy of MA and phylogenetic tree reconstruction. We evaluated performance by comparing the results to multiple sequence alignments with complete DNA sequences supplied as input. Genomic sequences of *Acinetobacter baumannii* strains were selected for *in silico* digestion to produce simulated genomes that mimic the assemblies of optical maps. For analysis of accuracy, the MA of these simulated genomes based on optical mapping patterns was compared with their respective multiple sequence alignment as the gold standard. We used 2 measures—precision and recall (see Methods for definitions)—to evaluate the performance. Our MA method for error-free genomes shows highly accurate results, with precision of 93.1% and recall of 93.7%. The precision and recall remain high upon introduction of missing sites and extra sites in various error rates. For disruptive errors including segmental insertion and segmental deletion, the precision and recall remains high until the error rate reaches a level so high that would not be seen in real data (Fig. S1).

Next, we evaluated the phylogenetic tree reconstruction method based on the MA results of OMMA. We assessed the accuracy of the reconstruction method using 100 sets of 32 simulated genomes generated by the introduction of accumulated mutations according to a virtual phylogeny of these genomes (see Methods for details). Based on the locations of the nicking sites, a simulated optical map was generated for each of these 32 genomes, and OMMA was used to form an MA. The phylogenetic tree of the 32 simulated genomes was then reconstructed using the unweighted pair-group method with an arithmetic mean approach based on the similarities among the genomes suggested by OMMA results (Fig. S2; see Methods for details). The overall accuracy of phylogenetic tree reconstruction was 96.5% or 99.4% with or without errors introduced, respectively, which indicates that the reconstruction method was accurate.

### Computational resources analysis

We separated the pipeline into pairwise alignment and MA for computational resources analysis in *A. baumannii, Escherichia coli*,and *Saccharomyces cerevisiae*. Pairwise alignment takes longer and more memory to run, while the MA requires fewer computational resources (Fig. S3-5). As the number of genomes increases, the number of pairwise alignments increases exponentially, and hence the number of segment links also increases exponentially. Therefore, CPU time and memory usage grows exponentially in both pairwise alignment and the MA process.

### OMMA captured and characterized complex genomic variations

OMMA provided a holistic view of the occurrence of complex genetic variations in various samples. To demonstrate the superiority of MA over pairwise alignment in the characterization of complex variations, we studied 3 types of regions in the human genome: regions with multiple haplotypes at the population level, regions with copy number variations (CNVs), and novel genomic regions that are not found in the reference human genome. We assembled optical map contigs of 154 human individuals from 26 human populations or 5 super-populations to identify these 3 types of complex regions [12]. We used OMMA to perform MA of these contigs with part of the reference genome hg38 used as one of the queries to obtain annotations related to the variations The use of hg38 is only for annotation purposes, and its presence does not affect the MA results, as shown in Fig. S6. We now discuss the study of the 3 types of regions as follows.

#### Regions with multiple haplotypes at the population level

**Figure 2:**
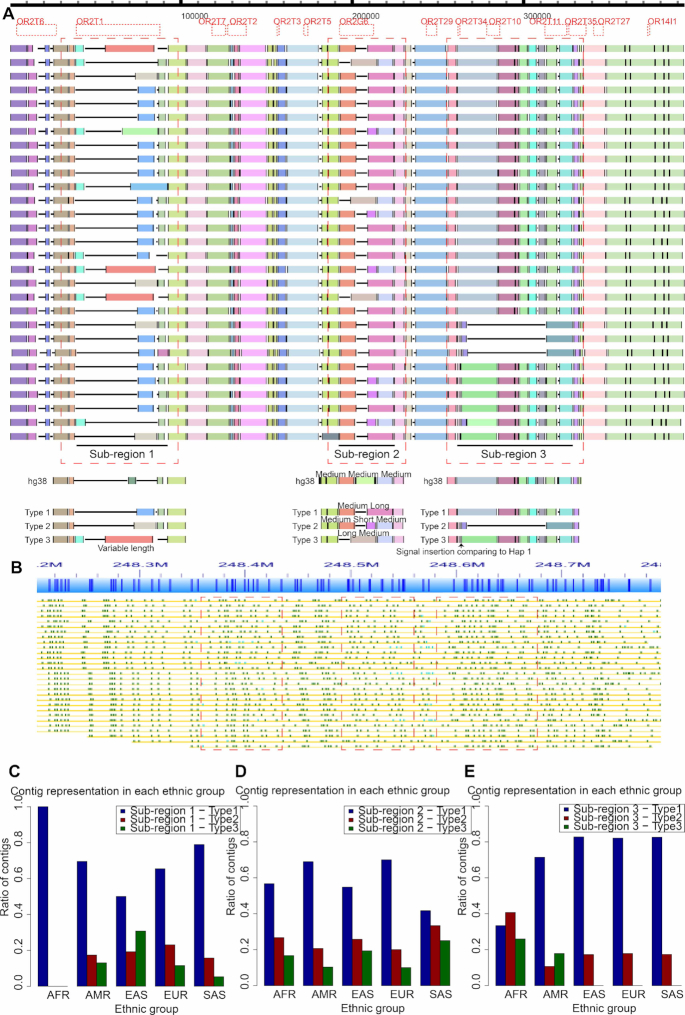
Multiple indels at the olfactory receptor (OR) region (1q44). (A) Three subregions that overlapped olfactory receptor genes with multiple variations were characterized. The multiple alignment shows the patterns of hg38 and major haplotypes of other contigs for each subregion. Only American contigs are shown for illustration purposes. Each row represents a contig. The MA of the contigs from all populations is shown in Fig. S7. (B) Alignment of optical maps from the American contigs on chromosome 1 was visualized using IrysView. The corresponding subregions shown in A are highlighted. It is noticeably difficult to resolve and characterize the labeling patterns in the presence of multiple haplotypes. (C–E) Contig representation at Subregion 1–3. In Subregion 1 (C), the African contigs only contained the Type 1 haplotype. In Subregion 2 (D), the contig ratio was similar among the various populations. In Subregion 3 (E), only the African and American contigs had the Type 3 haplotype. The African contigs also had more Type 2 haplotypes than the other populations. Abbreviations for the super-population code are as follows: AFR, African; AMR, Ad-mixed American; EAS, East Asian; EUR, European; SAS, South Asian.

Pairwise alignment only provides evidence for the presence or absence of variations from a reference, without considering the different labeling patterns contained in the queries. MA, however, provides a summary of all haplotypes present in the region. We illustrated the improved clarity of MA using OMMA at chromosome 1p44, which contains the olfactory receptor genes (Fig. 2A), compared with the traditional reference-based alignment view in IrysView (Fig. 2B). These genes are known to contain many small deletions and duplications [13]. The haplotype differences in this region are described separately in 3 subregions.

The first subregion overlapped the gene olfactory receptor family 2 subfamily T member 1 (*OR2T1*). There are various haplotypes, including 3 major haplotypes that we denote as Types 1, 2, and 3, of which the Type 1 haplotype was the most abundant (70.8%). Interestingly, the African contigs contained no Type 2 or Type 3 haplotypes (Fig. 2C). The second subregion contained the gene olfactory receptor family 2 subfamily G member 6 (*OR2G6*). Although hg38 was composed of a segment pattern to which we refer as medium-medium-medium (Fig. 2A; Subregion 2) for the middle 3 MA-blocks at this region, the contigs from various human populations actually reflected 3 segment patterns: long-medium, medium-long, and medium-short-medium. The Type 1 haplotype (59.0%) was more dominant than the Type 2 (25.0%) and Type 3 (16.0%) haplotypes (Fig. 2D). The third subregion spanned 4 genes (*OR2T34, OR2T10, OR2T11*, and *OR2T35*). The Type 1 and Type 3 haplotypes differed by only a single label. Despite this minor difference, only the contigs from the African and American populations had the Type 3 haplotype. The Type 2 haplotype was a deletion from the other types. The African contigs had a greater abundance (40.7%) of the Type 2 haplotype than all other populations (15.7%) (Fig. 2E). We also observed relationships in the haplotype distribution across 2 subregions. For example, Type 2 haplotypes in Subregion 3 are more likely to be followed by the Type 1 haplotype (30.5%) than by the Type 2 (3.1%) and Type 3 (9.5%) haplotypes in Subregion 2.

#### Copy number variations

**Figure 3:**
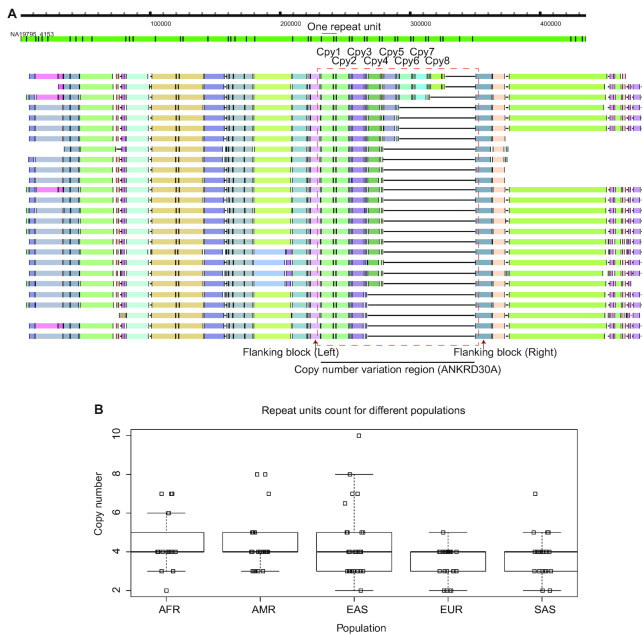
Copy number variation within the gene *ANKRD30A*. (A) Top: Example contig that contained the target CNV. Bottom: Multiple alignment of contigs from the American population offered a direct view of the whole CNV region. The number of copies in each contig was visualized directly, with each copy containing 2 labels. Only American contigs are shown for illustration purposes. The MA of contigs from all populations is shown in Fig. S8. (B) CNVs at *ANKRD30A* across various populations. The box plot summarizes the copy number observed across different populations, with each dot represents a copy number in an individual. In general, Europeans exhibited fewer copy numbers.

OMMA not only allowed direct visualization of the presence of CNVs; it also enabled the deduction of the exact copy number of the query. In the second complex case, we illustrated the characterization of CNV based on MA of contigs spanning the gene *ANKRD30A*, which contained tandem repeats including a very large repeat unit of size 11.1 kb [14]. The MA of contigs containing the CNV easily revealed the variable length of the contigs within this region, in comparison to adjacent conserved regions that had mostly the same length in the various contigs (Fig. 3A).

By choosing 2 flanking MA-blocks that corresponded to the boundary of the CNV region, the number of segments between them was used to deduce the number of copies in each contig. As an example, a contig from the sample NA19795 from the American population contains 16 alternating long and short segments. Because each repeat unit contains 2 segments (1 long and 1 short), we deduced that this contig has 8 copies. In comparison, the reference genome hg38 contains 8 segments, or 4 copies. The number of tandem repeat copies in other contigs ranged from 2 to 10. Based on the copy numbers in different contigs of each population, we investigated the correlation between copy number and ethnicity and found that European contigs had fewer copies than other populations (with marginal statistical significance; Tukey test, *P* = 0.06) (Fig. 3B).

#### Reconstruction of patterns in genomic regions not found in the reference

**Figure 4:**
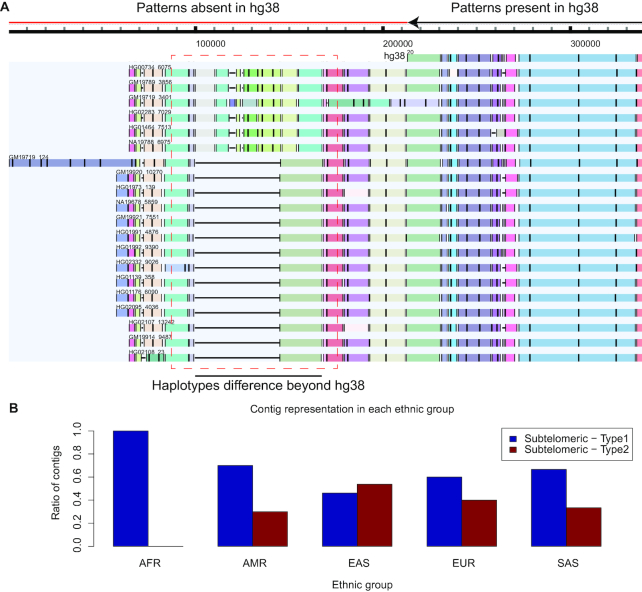
Subtelomeric region 20p. (A) Multiple alignment of the contigs from the American population that extend beyond the human reference hg38. The haplotype difference was visualized in a region not covered by hg38. Only American contigs are shown here for illustration purposes. The MA of the contigs from all populations is shown in Fig. S10. The labeling pattern from individual GM19921 beyond chromosome 20p was supported by pairwise alignment of optical maps (Fig. S9). (B) Contig representation of the 2 haplotypes in different populations at subtelomeric region 20p. Contigs from the African population were devoid of the Type 2 haplotype.

In the third case, we used MA to characterize the haplotype differences in the subtelomeric region of chromosome 20p, the sequence of which does not exist in the reference human genome. This region has been explored using optical maps and has been shown to display a pattern that is not found in the reference genome [15]. In our analysis, the extension of MA of the contigs beyond the sequence-containing portion of the reference chromosome 20p allowed us to discover a large indel (Type 1: without inserted pattern; Type 2: with inserted pattern) as a major haplotype difference among the contigs (Fig. 4A). The reliability of this extension is confirmed by alignment of molecules (Fig. S9). Notably, the African contigs contained only the Type 1 haplotype, whereas the contigs from other populations contained both haplotypes (Fig.   4B).

### OMMA revealed conservation of genomic structures and predicted colicin and bacteriophage integration

**Figure 5:**
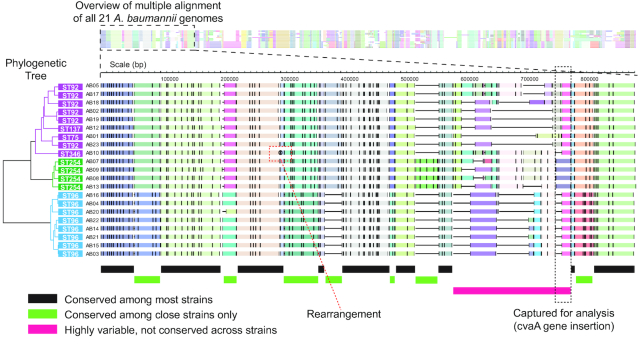
Overview of multiple alignment of 21 *A. baumannii* genomes. In the MA, the rectangles of various colors across a row represent segments from a query optical map. To indicate the alignment relationship of these segments, the segments in the same MA-block share a color and are arranged in the same column. Neighboring MA-blocks also share a color if they involve segments from the same set of optical maps. Black horizontal lines indicate empty spaces, and black circles indicate rearrangements in the MAs. A portion of the MA is magnified to demonstrate the division of genome regions into (1) those conserved among most strains, (2) those conserved only among close strains, and (3) highly variable regions that are not conserved across strains. An example of the captured region indicates the integration of Colicin V secretion genes. Another example of bacteriophage gene integration is shown in Fig. S11, which further describes the rearrangement event. The phylogenetic tree on the left was reconstructed on the basis of the OMMA multiple alignment of the optical maps of the 21 *A. baumannii* strains. The strains were separated into 2 major clusters of 13 and 8 strains, and the larger cluster was further divided into clusters of 9 and 4 strains.

We applied OMMA to optical maps generated from 21 drug-resistant *A. baumannii* genomes of various strains (Fig. 5). Briefly, the optical mapping data were generated and assembled into a single consensus optical mapping assembly for each of the 21 samples. OMMA combined the 8,315 segments from all strains into 823 MA-blocks.

The different regions in the MA could be roughly divided into 3 categories: (1) conserved among most strains, (2) conserved among close strains only, and (3) highly variable and not conserved across strains (Fig. 5). Although the segments shared by most strains likely represented the evolutionarily conserved regions, the segments shared only among the close strains could be the key to separation among the clusters of strains. The highly variable segments, in contrast, could be hot spots for the integration of genomic islands.

The categories could be partially supported by different conservation levels of the MA-blocks. Most MA-blocks were conserved, and 23.8% of MA-blocks contained segments from all genomes, which accounted for 49.5% of all segments (Fig. S12). These mainly constitute the region in category 1. Notably, some MA-blocks were evolutionarily conserved only within a certain set of genomes, as shown by the major peaks of MA-blocks with 8 (12.1%) and 13 (12.8%) segments, that constitute the region in category 2. This local conservation is likely the result of our experimental strains that were distributed into 2 main groups with 8 and 13 strains, respectively. (See the phylogenetic tree analysis section below.) The remaining MA-blocks that are not conserved constitute the region in category 3.

Segments that were not completely conserved that involved size changes were of greater interest than those with only label additions or deletions because the former corresponded to the indel of a large piece of DNA, which would be more likely associated with the gain or loss of an entire set of functional elements, whereas the latter would be associated with the introduction or disruption of a single functional element due to single-nucleotide variations or small indels. We deduced the identity of these functional elements by combining sequencing and optical mapping data (Fig. 5). For example, a potential insertion was likely the integration of a 9,286-bp sequence, including the Colicin V secretion gene *cvaA*. Another example was the integration of a 40,243-bp sequence, including several bacteriophage genes.

### OMMA-based phylogenetic tree reconstruction revealed an evolutionary relationship among strains

Next, we applied our phylogenetic tree reconstruction method to reconstruct the phylogenetic tree of the 21 strains of *A. baumannii* based on their OMMA alignment (Fig. 5). The reconstructed tree was separated into 2 major clusters of 13 and 8 strains. The larger cluster was further separated into 2 subclusters of 9 and 4 strains, in line with their respective multilocus sequence typing (MLST) information. Specifically, the tree from optical mapping separated the samples into 3 clusters of sequence types (STs): (1) [ST75, ST92, ST137, ST346], (2) [ST254], and (3) [ST96]. In fact, the 4 STs in the first cluster were highly similar and differed only in the single-nucleotide polymorphisms of a single gene.

One advantage of our method is that it separates strains on the basis of whole-genome structures, unlike the traditional MLST method, which relies on only a selected set of genes. As a result, our method provides substantially greater detail about the evolutionary relationships among the strains. For example, the strains classified as ST96 by MLST could be further divided into multiple groups based on our results.

## Discussion

Our OMMA method combines multiple queries into a single comparison, which assists with a wide range of analyses, including those in comparative genomics and population genomics. Multiple alignment provides a comprehensive view of the comparison of queries. With variations captured across different queries, our method is able to reflect the level of variability or conservation within a certain region and thus locate potential hot spots for genomic variations.

OMMA is also a useful tool for population genomics analyses. Variable haplotypes can be classified quickly, and their relative abundance in various populations can then be directly visualized and analyzed. With the phylogenetic tree reconstructed, the elements responsible for the differentiation into clusters can also be determined.

One major advantage of the use of optical mapping over sequencing is its ability to directly visualize and study the genome structure. The assembled optical mapping contigs are usually much longer than sequence assemblies, which usually break into pieces at short repetitive regions. It also becomes more challenging when we need to characterize complex structural changes in larger genomes like the human genome by examining sequence assembly.

The OMMA pipeline offers great flexibility in customizing procedures. The flexibility of this method allows the task of MA of a very large genome to be divided into smaller jobs. The results can then be combined into a single MA during the final step. Our method also supports aligning queries with rearrangements including inversions and intrachromosomal translocations. In addition to the applications demonstrated here, our MA method could be extended for other studies, such as pan-genome construction and genomic island prediction. Our method has limitations, including a low tolerance to very large segmental duplications (e.g., 250 kb) with multiple copies. However, with the recently launched direct label and stain (DLS) chemistry that no longer introduces double-strand DNA breaks at 2 closely located nicks, the improved quality of assemblies should reduce the effect of this limitation on MA.

## Conclusions

In this paper, we describe the first rearrangement-tolerant and nonproprietary MA method for optical mapping of data. The method's accuracy was assessed using an *A. baumannii* dataset with high precision and recall. We demonstrate the application of the MA results to phylogenetic analyses and complex region analyses (e.g., multiple haplotypes, CNVs, and novel genomic region). OMMA could serve as a fundamental tool for the further development of more specific analytic methods for the study of comparative and population genomics.

## Methods

### OMMA overview

The OMMA program was developed to compute segment-matching information to generate a series of MA-blocks as a collection of matching segments of entries. The entire pipeline is separated into a preparation step and an MA step that can be further divided into 3 main substeps (modules) (Fig. 1). In the first substep, the matching segments from pairwise alignments or other sources are used to construct the blocks. In the second substep, these blocks are sorted to minimize rearrangement events. Finally, in the third substep, proximal segments are merged if their sizes are similar.

### Preparation step

At the preparation step before the core modules are run in MA, we must generate some clues about which segments on different queries should be put together. Usually, this process considers not only the size matching of a pair of target segments alone but also accounts for the size matching of their proximate segments. We define a piece of evidence that helps to determine the matching of segments as a "source." A source is composed of a set of "segment links" that are denoted as a matching pair of segments from different queries. Our method accepts 2 sources that provide clues about the segment links based on similar labeling patterns: pairwise alignment among queries and the MA results.

#### Pairwise alignment result as sources

All queries are aligned in a pairwise manner (Fig. 1B), followed by derivation of the segment links from the alignments of each pair of queries. The pairwise alignment results were generated by OMBlast [5], which could output partial alignments (local alignments between a pair of queries) that are critical to MA of regions with rearrangement.

#### Multiple alignment result as a source

The MA result (generated by the OMMA pipeline) can also be used as a source because the results are intuitively a collection of matching segments. The result is particularly useful in large-scale MA, such as whole-genome MA in humans, for which 1-step MA is not computationally feasible. By dividing the large number of queries into separate subtasks, several MAs on a smaller scale can be achieved, and the results can serve as the sources for a global MA.

### MA-block construction

#### Construction of MA-blocks based on segment links

For MA of *Q* queries, each MA-block is represented by a binary vector of length *Q*, where a “non-empty” entry or “1” indicates that a query participates in this block, and an “empty” entry or “0” indicates that it does not. A non-empty entry can be occupied by only 1 segment from the query. No more than 1 segment from the same query can be assigned to each MA-block.

An undirected graph is constructed with the segments as vertices and the segment links as edges (Fig. 1C). Each connected component is treated as an MA-block "candidate." A "valid" MA-block is defined as a collection of segments with ≤1 segment from the same query. If an MA-block candidate fulfills this criterion, all segments in the candidate are directly converted into an MA-block. Otherwise, each individual segment in the candidate is instead assigned to a separate MA-block.

#### Segment links from multiple sources

The selection of a proper set of parameters in pairwise alignment is difficult. To solve this problem, segment links from various sources can be supplied in the MA processes. Briefly, segment links from the most confident sources (such as pairwise alignment results with more stringent parameters) are first used to construct MA-blocks. The segment links from less confident sources (such as pairwise alignment results with more lenient parameters) are then used to connect the MA-blocks. Two connected MA-blocks are merged if their binary vector does not overlap (i.e., if they do not have segments from the same queries after merging).

### MA-block sorting

The MA-blocks are sorted to better illustrate the overall pattern of the queries. Here, we briefly demonstrate the idea using Fig. 1D before discussing the details of implementation. With different MA-blocks generated from the previous step, it is obvious to place MA-block [A1, B1, C1] before MA-block [A2, B2, C2] (i.e., segment A1 is followed by segment A2 on query A, and the same applied to query B and C) because the segments from all queries in these 2 blocks are consecutive. However, the problem becomes more complicated after rearrangement such as inversion or translocation. For example, we must choose what follows the MA-block [A3, B3, C3]; it could be MA-block [A4, C8] (i.e., because segment A3 is followed by segment A4 on query A), MA-block [B4] (i.e., because segment B3 is followed by segment B4 on query B), or MA-block [A8, B9, C4] (i.e., because segment C3 is followed by segment C4 on query C).

More formally, the MA-blocks are sorted to minimize the number of rearrangement events and maximize the matching events. For *B* MA-blocks constructed from the previous module, consider a non-empty *q*th entry in the *b*th MA-block and a non-empty *q*th entry in the (*b* +*x*)th MA-block, where *x* is a positive integer and any *q*th entries are empty in the (*b* + 1)th to (*b*+*x*− 1)th MA-blocks. A "matching event" occurs if the 2 entries represent consecutive segments in the original *q*th query and have the same orientation. In contrast, a "rearrangement event" occurs when the 2 entries are in different orientations or if they are not consecutive segments ordered in the original *q*th query. Note that the sum of matching and rearrangement events in the final chain remains constant for the same set of queries. This problem is equivalent to a nondeterministic polynomial-time hard (NP-hard) traveling salesman problem, in which all vertices (MA-blocks) must be traversed exactly once, except that no limitation is set on the start and end points. Because determination of an optimized solution is computationally intensive, the nearest neighbor–joining algorithm was devised to approximate a suboptimal solution.

#### Nearest neighbor–joining algorithm

In the nearest neighbor–joining algorithm, the MA-blocks are connected into multiple chains. The goal is to repeatedly connect chains until a single chain remains as the solution to the order of the MA-blocks. In the beginning, 1 chain is created for each MA-block, resulting in *C* chains. The connection candidates from each pair of chains are added into a priority queue. In each round of connection, a pair of chains that results in the highest connection priority is pulled from the queue and connected to a new chain, followed by an update on the connection candidates for the new chain. The connection process is repeated for *C* − 1 rounds to connect all *C* chains into 1 single chain.

#### Connection priority for 2 chains of MA-blocks

The connection priority for 2 chains of MA-blocks is based entirely on the relationship of the entries in the 2 chains. In total, *Q* relationships are built on the basis of *Q* sets of entries compared within the same query. Only the last non-empty entry from the former chain and first non-empty entry from the latter chain are compared. We define the relationship of the entries from a particular query as matching and rearrangement if the 2 selected entries are consecutive and nonconsecutive segments, respectively. In addition to matching, if the 2 entries are taken from the last MA-block of the former chain and first MA-block of the latter chain, they are further categorized as direct matching. The relationship is set as empty if no non-empty entry exists in either chain. An example is shown in Fig. S13, in which the relationships of the 4 sets of entries from queries A, B, C, and D represent matching, direct matching, rearrangement, and empty relationships, respectively. The parameters are defined for the count of relationships between the 2 chains as follows:

- *n_m_*: matching (note that a direct matching relationship is also counted here)
- *n_d_*: direct matching
- *n_r_*: rearrangement
- *n*_rr_: reference rearrangement (if reference is provided by the user)
- *n_e_*: empty

The parameters above always meet the following criteria:

- ${n_m} + {n_r} + {n_e} = Q$
- ${n_d} \le {n_m}$
- *n*_rr_ ≤ *n_r_*

The connection priority for a pair of chains is listed below:

1. At least 1 matching relationship exists (${n_m} \ge 1$).
2. No rearrangement is found (${n_r} = 0$).
3. If no rearrangement is found, *n_d_* is maximized, followed by maximizing *n_m_*.
4. If rearrangement is found,
  - the connection results in no rearrangement in reference (*n*_rr_ = 0, if reference is provided by the user).
  - *n_r_* is minimized, followed by maximizing *n_m_*, followed by maximizing *n_d_*.

### Directed graph representation for multiple alignment

The MA results can be represented as an acyclic directed graph. Consider an acyclic directed graph constructed with MA-blocks as the vertices. A directed edge from MA-block *b* to *b*+ *x* is built if 2 non-empty entries exist from the same query *q* in *b*th and (*b* +*x*)th MA-blocks, where *x* is a positive integer and all entries from query *q* are empty in the (*b* + 1)th to (*b* +*x* − 1)th MA-blocks. An edge is built between 2 MA-blocks with a weight equal to the sum of the matching and rearrangement relationships. Its direction follows the order of the MA-blocks. Such a representation simplifies the interpretation of the actual variation and noise, which can be removed by filtering the edges with minimum weight.

### Merging

We wish to merge MA-blocks without introducing new rearrangements. The order of the MA-blocks other than the merging target remains unchanged in the merging actions. With such a constraint, the merging step can effectively increase the sensitivity of the final MA result. From the directed graph representation of the sorted MA-blocks, the merging of 2 MA-blocks leads to new rearrangements if the set of descendant vertices of 1 MA-block intersects the set of ancestor vertices of another MA-block.

#### Merging of MA-blocks by proximity

Consider a directed graph representation of the sorted MA-blocks (Fig. 1E), with MA-blocks as vertices. Two MA-blocks that share ≥1 incoming neighbor or ≥1 outgoing neighbor are defined as proximate. Two proximate MA-blocks are merged if the average size of the segments in 1 MA-block matches those in the other MA-block. The merging of 2 MA-blocks can be disruptive and lead to rearrangement. To avoid the introduction of new rearrangements, we must check for overlapping of the ancestor and descendant vertices. The ancestor and descendant vertices of an MA-block are obtained by traversing the directed graph, and the ancestor and descendant vertices are updated by dynamic programming. After each merging step, the directed graph and the ancestor and descendant vertices are updated.

#### Merging of MA-blocks by segment links

Another merging strategy relies on segment links, as described above in the MA-block construction step. This time, the connections are taken as evidence for the potential merging of 2 MA-blocks that include 2 segments that form a connection. Like merging proximate MA-blocks, the checking and update steps described in the previous section are used to prevent the introduction of rearrangements during the merging of 2 connected MA-blocks.

### Assessment of accuracy of OMMA

Because there is no standard answer for MA based merely on optical mapping patterns, the accuracy of the present method was inferred from the consistency attained among MAs from optical mapping and MAs from sequencing. MA software based on genomic sequences was used to assess the accuracy of MA in optical mapping. In this study, the multiple sequence alignment was performed using Mugsy v1r2.3 [16], which used results from MUMmer 3.20 [17] under the default parameters.

The accuracy was measured by 2 parameters, precision and recall. In MA of optical mapping, a segment *i* forms segment-pair *p_i_,_j_* with another segment *j* for $i \ne j$ if the 2 segments belong to the same MA-block. In MA from sequencing, consider a segment *m* of length *l_m_*, a segment *n* of length *l_n_*, and the length of multiply aligned sequence *l_seq_*. The similarity *s_m_,_n_* of segment *m* to segment *n* is defined as *l_seq_*/*l_m_*. The segment *m* forms segment-pair *p_m_,_n_* with segment *n* if:

\begin{equation*}
{s_{m,n}} \ge 0.8\,\,{\rm{and}}\,\,0.8 \le \frac{{{l_m}}}{{{l_n}}} \le 1.25.
\end{equation*}

An intersected segment-pair set was created by the intersection of a segment-pair set derived from the MA of optical mapping and one derived from MA of sequence. Precision was calculated as the number of intersected segment-pairs divided by the number of segment-pairs derived from the MA of optical mapping. Recall was calculated as the number of intersected segment-pairs divided by the number of segment-pairs derived from the MA of sequence.

To test the performance of MAs on error-containing queries, 4 types of errors (extra sites, missing sites, segmental insertions, and segmental deletions) were introduced at different levels. Extra sites error was simulated by adding nicking sites at a random location. Missing sites error was simulated by randomly removing the existing nicking sites. Segmental insertions and deletions errors were simulated by adding or deleting a fragment at a random location. The size of the deleted or inserted fragment ranged from 1 bp to 10 kb, with the size following the ${{\rm{\chi }}^2}$ distribution (degrees of freedom = 1). Rates of all 4 types of errors ranged from 0 to a very high value such that it is not seen in typical optical mapping data. To assess the effects of each error type, only 1 type of error was introduced at a time.

### Phylogenetic tree reconstruction

Our reconstruction method is based mainly on the assumption that 2 strains have greater similarity if they share more MA-blocks in the MA. From the MA, the distance of sample *i* relative to sample *j* is defined as follows:

\begin{equation*}
{d_{ij}} = 1 - \displaystyle\frac{{{{f_{ij}}}}/{{{f_{jj}}}} + {{f_{ji}}}/{{f_{ii}}}}2.
\end{equation*}

where *f_ij_* represents the number of MA-blocks shared by *i* and *j*. Note that ${f_{ii}} \ge {f_{ij}}$, ${f_{ij}} = {f_{ji}}$, and ${d_{ij}} = {d_{ji}}$.

A distance matrix was built to reconstruct the phylogenetic tree via an unweighted pair-group method with an arithmetic mean approach. The tree was then visualized with the Analyses of Phylogenetics and Evolution (APE) package in R [18].

#### Generation of simulated genomes for phylogenetic analysis

The performance of our method of phylogenetic analysis was assessed using simulated genomes without any error, or with missing site rate of 7e−3, extra site rate of 1e−6, deletion rate of 1e−5, and insertion rate of 1e−5. The virtual genomes were simulated according to a virtual phylogeny in Fig. S14. Briefly, in the first generation, random sequence mutations were introduced into an ancestor genome using the profile-based Illumina pair-end reads simulator (pIRS) program [19] to simulate 2 children genomes. These genomes were then taken as the parent genomes in the next generation to synthesize more children genomes. At the *n*th generation, 2*^n^* children genomes were simulated. Only the children genomes at the last generation were used as the input of OMMA.

#### Assessment of phylogenetic tree accuracy

Based on the generation method, offspring of a parent genome (2^*k*^ children genomes for a parent genome at the *(n − k + 1)*th generation, where *n* is the total number of generations) should be classified in the same clade. The individual accuracy of each phylogenetic tree was defined as the ratio of clades fulfilling the above classification. The overall accuracy was then calculated as the average accuracy of all phylogenetic trees from 100 sets of simulated genomes.

### Generation of optical mapping and sequencing data

#### Bacteria sample collection

All 21 isolates were sampled from inpatient specimens or colonization studies in children for *A. baumannii*. The isolates selected for this project had been independently characterized by pulsed-field gel electrophoresis and/or MLST. The results were unknown to the optical mapping analysis procedure.

#### Genomic DNA extraction

Megabase-sized bacterial genomic DNA was extracted with the agarose gel plug method. Embedment in the porous agarose matrix protected the DNA from physical shearing while allowing access to restriction enzymes. The bacterial cell pellets were resuspended and embedded in low-melting agarose to form gel plugs. The plugs were treated with proteinase K and lysozyme for cell lysis. After washing several times in 1× Tris-EDTA buffer, the plugs were melted at 70°C for 2 min and equilibrated at 42°C for 5 min before Gelase (Epicentre, Madison, WI) was added to solubilize the sample. The solubilized DNA obtained was concentrated by drop-dialysis with 1× Tris-EDTA buffer for 2.5 h at room temperature. The high–molecular weight DNA samples were then quantified with a Quant-iT dsDNA Assay Kit (Thermo Fisher Scientific, Walthan, MA). The DNA quality was checked by contour-clamped homogenous electric field gel electrophoresis.

#### Nicking, labeling, and repairing reactions

The bacterial DNA samples were nicked, labeled, repaired, and stained. In summary, the single-strand breaks (nicks) were introduced to 300 ng bacterial DNA by nicking endonuclease Nt.BspQI (New England Biolabs) at 37°C for 2 h. The DNA nicks were filled with fluorescent nucleotides by Taq polymerase and sealed with Taq DNA ligase (New England Biolabs, Ipswich, MA). The backbone of the double-stranded DNA was stained overnight with fluorescence dye YOYO-1 (Invitrogen, Carlsbad, CA).

#### Imaging and raw data processing

The stained double-stranded DNA with fluorescent labels was loaded by electric current onto a chip that contained massively parallel nanochannel arrays, upon which the DNA was linearized and imaged. The lengths and relative positions of the fluorescent labels of the DNA molecules were calculated from the images to individual single-molecule maps (optical maps) by estimating any errors in size scaling and missing or spurious labels.

#### Optical map assembly

The optical mapping assembly of the genome of *A. baumannii* was performed using the standard pipeline in “Bionano Solve 3.1” [7], followed by a custom refinement script to trim the contigs. The plasmids and incomplete genomes were removed from further analysis. Because bacterial genomes are circular, for ease of analysis and visualization, all genomes were oriented such that they all began with a conserved pattern across most *A. baumannii* strains.

#### Sequencing data generation and sequence assembly

To reveal the identity of the variations detected by optical mapping, 6 *A. baumannii* samples were selected for sequencing and assembled using Short Oligonucleotide Analysis Package (SOAPdenovo) [20]. The assembled contigs were annotated using Prokka [21]. To deduce annotations on optical mapping contigs, the assembled sequence contigs were aligned on the optical mapping contigs using OMBlast [5], with annotations assigned on the relative aligned position on the optical mapping contigs.

#### Human optical maps data for validation

The generation of assembled contigs of 154 human individuals from 5 super-populations (African, ad-mixed American, East Asian, European, South Asia) used for characterization of complex genomic variations was reported by Levi-Sakin et al. [12].

## Availability of supporting source code and requirements

Project name: OMTools Project

Project home page: https://github.com/TF-Chan-Lab/OMTools

Operating system: Platform independent

Programming language: Java

Other requirements: Java 8 or higher

License: GNU GPL

The OMMA is a new module in the OMTools package as published by Leung et al. [22]. The resource has been submitted to SciCrunch.org with the RRID:SCR_017143.

## Availability of supporting data and materials

The raw optical mapping and sequencing data of the *A. baumannii* strains are available at NCBI (BioProject PRJNA486415). Other data further supporting this work can be found in the *GigaScience* repository, GigaDB [23].

## Additional files

Figure S1: The performance of multiple alignment on genomes with various types of errors at different rates

Figure S2: Phylogenetic tree reconstructed based on multiple alignment by OMMA of simulated genomes

Figure S3: CPU time and memory usage for pairwise alignment and multiple alignment of different number of A. baumannii genomes

Figure S4: CPU time and memory usage for pairwise alignment and multiple alignment of different number of E. coli genomes

Figure S5: CPU time and memory usage for pairwise alignment and multiple alignment of different number of S. cerevisiae genomes

Figure S6: Multiple alignment by OMMA of the contigs with (A) and without (B) hg38 of Figure 2 using the same parameters

Figure S7: Multiple alignment by OMMA of the contigs from all populations at the olfactory receptor region (1q44)

Figure S8: Multiple alignment of contigs by OMMA from all populations at gene ANKRD30A

Figure S9: Alignment of optical maps from individual GM19921 on chromosome 20 using IrysView

Figure S10: Multiple alignment of the contigs by OMMA from all populations at the subtelomeric region of chromosome 20p

Figure S11: Example of genome rearrangement

Figure S12: Occurrence of segments

Figure S13: An example showing the connection of two chains of blocks

Figure S14: Data simulation for phylogenetic tree assessment

## Abbreviations

bp: base pairs; CNV: copy number variation; CPU: central processing unit; CRISPR: clustered regularly interspaced short palindromic repeats; DLS: direct label and stain; EDTA: ethylenediaminetetraacetic acid; kb: kilobase pairs; MA: multiple alignment; Mb: megabase pairs; MLST: multilocus sequence typing; NCBI: National Center for Biotechnology Information; OMMA: optical mapping by multiple alignment; ST: sequence type.

## Competing interests

The authors declare that they have no competing interests.

## Funding

T.F.C., K.Y.Y., and P.L.H. are partially supported by a Health and Medical Research Fund (HMRF12110542) from the Food and Health Bureau of the Hong Kong Special Administrative Region (HKSAR). A.K.Y.L. and T.F.C. are partially supported by the CUHK Direct Grants 3132782, 4053242, and 4053364, a General Research Fund (14102014), Collaborative Research Fund (C4042-14G), an Area of Excellence Scheme (AoE/M-403/16) from the HKSAR Research Grants Council, and funding from the Innovation and Technology Commission, Hong Kong Government to the State Key Laboratory.

## Authors' contributions

T.F.C. and K.Y.Y. conceived the study. A.K.L., T.F.C., and K.Y.Y. designed the computational methods. A.K.L. and L.L. implemented the computational methods. M.C.J.L. and P.L.H. provided the DNA from *A. baumannii* samples and conducted the MLST and pulsed-field gel electrophoresis experiment. Y.Y.L., C.C., and P.Y.K. produced the optical mapping data of *A. baumannii*. A.K.L. processed the optical mapping data. AKL, T.F.C., and K.Y.Y. wrote the manuscript. All authors read and approved the final manuscript. The authors wish to acknowledge Allen Yu and Aldrin Yim from Codex Genetics for offering initilal technical support during the course of this work.

## Supplementary Material

giz079_GIGA-D-18-00292_Original_Submission

giz079_GIGA-D-18-00292_R2

giz079_GIGA-D-18-00292_Revision_1

giz079_Response_to_Reviewer_Comments_Original_Submission

giz079_Response_to_Reviewer_Comments_Revision_1

giz079_Reviewer_1_Report_Original_Submission Christina Boucher -- 9/1/2018 Reviewed

giz079_Reviewer_1_Report_Revision_1 Christina Boucher -- 1/28/2019 Reviewed

giz079_Reviewer_1_Report_Revision_2 Christina Boucher -- 5/13/2019 Reviewed

giz079_Reviewer_2_Report_Original_Submission Jay Ghurye -- 10/11/2018 Reviewed

giz079_Reviewer_2_Report_Revision_1 Jay Ghurye -- 1/21/2019 Reviewed

giz079_Supplemental_File
