# Supplementary material for: OMMA enables population-scale analysis of complex genomic features and phylogenomic relationships from nanochannel-based optical maps
Source: Gigascience. 2019 Jul 9;8(7):giz079. doi: 10.1093/gigascience/giz079 (PMC6615982; doi:10.1093/gigascience/giz079)
Supplement: giz079_GIGA-D-18-00292_Original_Submission [file giz079_giga-d-18-00292_original_submission.pdf]

GigaScience

OMMA enables population-scale analysis of complex genomic features and phylogenomic relationships from nanochannel-based optical maps

--Manuscript Draft--

**Manuscript Number:**	GIGA-D-18-00292
**Full Title:**	OMMA enables population-scale analysis of complex genomic features and phylogenomic relationships from nanochannel-based optical maps
**Article Type:**	Technical Note
**Funding Information:**	Health and Medical Research Fund (12110542) — Dr. Ting-Fung Chan<br>Research Grants Council, University Grants Committee (14102014) — Dr. Ting-Fung Chan<br>Research Grants Council, University Grants Committee (C4042-14G) — Dr. Ting-Fung Chan<br>University Grants Committee (AoE/M-403/16) — Dr. Ting-Fung Chan<br>Innovation and Technology Commission - Hong Kong — Dr. Ting-Fung Chan
**Abstract:**	BACKGROUND<br><br>Optical mapping is an emerging technology that complements sequencing-based methods in genome analysis. It is widely used in improving genome assemblies and detecting structural variations by providing information over much longer (up to 1Mbp) reads. Current standards in optical mapping analysis involve assembling optical maps into contigs and aligning them to a reference, which is limited to pairwise comparison and becomes bias-prone when analyzing multiple samples. FINDINGS: We present a new method, OMMA, that extends optical mapping to the study of complex genomic features by simultaneously interrogating optical maps across many samples in a reference-independent manner. OMMA captures and characterizes complex genomic features, e.g. multiple haplotypes, copy-number variations, and subtelomeric structures when applying to 154 human samples across the 26 populations sequenced in the 1000 Genomes Project. For small genomes such as pathogenic bacteria, OMMA accurately reconstructs the phylogenomic relationships and identifies functional elements across 21 Acinetobacter baumannii strains. CONCLUSIONS: With the increasing data throughput of optical mapping system, the use of this technology in comparative genome analysis across many samples will become feasible. OMMA is a timely solution that can address such computational need. The OMMA software is available at https://github.com/TF-Chan-Lab/OMTools.
**Corresponding Author:**	TingFung Chan, PhD<br>Chinese University of Hong Kong<br>Shatin, HONG KONG
**Corresponding Author Secondary Information:**	
**Corresponding Author's Institution:**	Chinese University of Hong Kong
**Corresponding Author's Secondary Institution:**	
**First Author:**	Alden King-Yung Leung, PhD
**First Author Secondary Information:**	
**Order of Authors:**	Alden King-Yung Leung, PhD<br>Melissa Chun-Jiao Liu<br>Yvonne Yuk-Yin Lai, PhD

Powered by Editorial Manager® and ProduXion Manager® from Aries Systems Corporation

	Catherine Chu
	Pui-Yan Kwok, MD PhD
	Pak-Leung Ho
	Kevin Yuk-lap Yip, PhD
	Ting-Fung Chan, PhD
**Order of Authors Secondary Information:**	
**Additional Information:**	
**Question**	**Response**
Are you submitting this manuscript to a special series or article collection?	No
**Experimental design and statistics**<br><br>Full details of the experimental design and statistical methods used should be given in the Methods section, as detailed in our Minimum Standards Reporting Checklist. Information essential to interpreting the data presented should be made available in the figure legends.<br><br>Have you included all the information requested in your manuscript?	Yes
**Resources**<br><br>A description of all resources used, including antibodies, cell lines, animals and software tools, with enough information to allow them to be uniquely identified, should be included in the Methods section. Authors are strongly encouraged to cite Research Resource Identifiers (RRIDs) for antibodies, model organisms and tools, where possible.<br><br>Have you included the information requested as detailed in our Minimum Standards Reporting Checklist?	Yes
**Availability of data and materials**<br><br>All datasets and code on which the conclusions of the paper rely must be	Yes

either included in your submission or deposited in publicly available repositories (where available and ethically appropriate), referencing such data using a unique identifier in the references and in the "Availability of Data and Materials" section of your manuscript.<br><br>Have you have met the above requirement as detailed in our Minimum Standards Reporting Checklist?	

Powered by Editorial Manager® and ProduXion Manager® from Aries Systems Corporation

Click here to download Manuscript 20180803_OMMA_Manuscript_GigaScience_2.docx

OMMA enables population-scale analysis of complex genomic features and phylogenomic relationships from nanochannel-based optical maps

Alden King-Yung Leung[1], Melissa Chun-Jiao Liu[3], Yvonne Yuk-Yin Lai[4,7], Catherine Chu[4,7], Pui-Yan Kwok[4,7], Pak-Leung Ho[3], Kevin Yuk-lap Yip[2,6*], Ting-Fung Chan[1,5,6*]

[1]School of Life Sciences, [2]Department of Computer Science and Engineering, [5]Partner State Key Laboratory of Agrobiotechnology, and [6]Hong Kong Bioinformatics Centre, The Chinese University of Hong Kong, Shatin, Hong Kong

[3]Carol Yu Center for Infection and Department of Microbiology, The University of Hong Kong, Queen Mary Hospital, Pok Fu Lam, Hong Kong

[4]Cardiovascular Research Institute, and [7]Institute of Human Genetics, University of California, San Francisco, California 94153, USA.

* indicates corresponding authors

Abstract

BACKGROUND: Optical mapping is an emerging technology that complements sequencing-based methods in genome analysis. It is widely used in improving genome assemblies and detecting structural variations by providing information over much longer (up to 1Mbp) reads. Current standards in optical mapping analysis involve assembling optical maps into contigs and aligning them to a reference, which is limited to pairwise comparison and becomes bias-prone when analyzing multiple samples. FINDINGS: We present a new method, OMMA, that extends optical mapping to the study of complex genomic features by simultaneously interrogating optical maps across many samples in a reference-independent manner. OMMA captures and characterizes complex genomic features, e.g. multiple haplotypes, copy-number variations, and subtelomeric structures when applying to 154 human samples across the 26 populations sequenced in the 1000 Genomes Project. For small genomes such as pathogenic bacteria, OMMA accurately reconstructs the phylogenomic relationships and identifies functional elements across 21 *Acinetobacter baumannii* strains. CONCLUSIONS: With the increasing data throughput of optical mapping system, the use of this technology in comparative genome analysis across many samples will become feasible. OMMA is a timely solution that can address such computational need. The OMMA software is available at https://github.com/TF-Chan-Lab/OMTools.

Keywords

Optical mapping, comparative genomics, structural variation, copy-number variation, haplotypes, single-molecule analysis

Findings

Background

Optical mapping captures the labeling patterns of long DNA molecules and has become a complementary approach to sequencing-based methods [1]. DNA labels are usually created by a short but specific nicking restriction enzyme (nickase), but alternative labeling strategies, such as methylations [2-3] and sequence-specific labeling based on CRISPR technology [4] have also been described. With optical map ranges from 100 kbp to as high as 1 Mbp, optical mapping is well suited to assist with sequence scaffolding in genome assembly and in the detection of large structural variations.

Multiple alignment is a process in which multiple queries are aligned without relying upon a reference. This type of comparison is especially useful when a standard reference is not available or is of poor quality. Also, in certain variable regions, the alignment of multiple queries to a reference could only show a one-to-one difference between the individual query and the reference. In contrast, multiple alignment of these queries helps to differentiate groups of patterns among the queries. Unlike pairwise alignment, for which many algorithms have been designed [5-8], multiple alignment remains an underdeveloped method for optical mapping. The other available tool is included in the proprietary software Bionumerics v7 [9], but this method is not sensitive to genomic rearrangement and requires complete genomes as inputs.

We developed a novel multiple alignment algorithm for population-scale analysis of optical mapping data: optical mapping by multiple alignment (OMMA). We describe herein the algorithm and demonstrate its effectiveness with both simulated and experimental data. We also demonstrate how OMMA can be used to resolve complex genomic features and reconstruct phylogenomic relationships.

OMMA pipeline

The complete OMMA pipeline has two steps—the preparation step and the multiple alignment step (Figure 1). During the preparation step, optical maps (Figure 1A) are aligned in a pairwise manner (Figure 1B). Two segments from two different optical maps are said to match if their left and right labels are both aligned. In the multiple alignment step, information about the matching segments is used to produce chains of multiple alignment (MA)–blocks as the multiple alignment results; an MA-block is defined as a collection of segments, each from a different optical map, that all match each other. This step can be further divided into three main substeps (modules) (Figure 1C-E). In the first substep, MA-blocks are formed based on the results of the preparation step (Figure 1C). In the second substep, the MA-blocks are sorted to maximize matching and to minimize rearrangement events (Figure 1D). Finally, in the third substep, proximate MA-blocks that are similar to each other are merged (Figure 1E). The details of the pipeline are described in the Methods.

Performance analysis

We evaluated performance by comparing the results to multiple sequence alignments with complete DNA sequences supplied as input. Genomic sequences of *Acinetobacter baumannii* strains were selected for *in silico* digestion to produce simulated genomes that mimic the assemblies of optical maps. For analysis of accuracy, the multiple alignment of these simulated genomes based on optical mapping patterns was compared with their respective multiple sequence alignment as the gold standard. We used two measures—precision and recall (see Methods for definitions)—to evaluate the performance. Our multiple alignment method shows highly accurate results, with precision of 93.4% and recall of 92.6%.

Next, we evaluated the phylogenetic tree reconstruction method based on the multiple alignment results of OMMA. We assessed the accuracy of the reconstruction method using a set of 32 simulated genomes generated by the introduction of accumulated mutations according to a virtual phylogeny of these genomes (see Methods for details). Based on the locations of the nicking sites, a simulated optical map was generated for each of these 32 genomes, and OMMA was used to form a multiple alignment. The phylogenetic tree of the 32 simulated genomes was then reconstructed using the unweighted pair-group method with an arithmetic

mean approach based on the similarities among the genomes suggested by OMMA results (Figure S1; see Methods for details). This reconstructed tree has the same topology as the simulated tree, which indicates that the reconstruction method was accurate.

OMMA captured and characterized complex genomic variations

OMMA provided a holistic view of the occurrence of complex genetic variations in various samples. To demonstrate the superiority of multiple alignment over pairwise alignment in the characterization of complex variations, we studied three types of regions in the human genome: regions with multiple haplotypes at the population level, regions with copy number variations, and novel genomic regions that are not found in the reference human genome. We assembled optical map contigs of 154 human individuals from 26 human populations or five super-populations to identify these three types of complex regions (Levi-Sakin et al., manuscript under review). We used OMMA to perform multiple alignment of these contigs with part of the reference genome hg38 used as one of the queries to obtain a better biological interpretation of the variations. We now discuss the study of the three types of regions in turn.

Regions with multiple haplotypes at the population level

Pairwise alignment only provides evidence for the presence or absence of variations from a reference, without considering the different labeling patterns contained in the queries. Multiple alignment, however, provides a better summary of all haplotypes present in the region. We illustrated the improved clarity of multiple alignment using OMMA at chromosome 1p44, which contains the olfactory receptor genes (Figure 2A), compared with the traditional reference-based alignment view in IrysView (Figure 2B). These genes are known to contain many small deletions and duplications [10]. The haplotype differences in this region are described separately in three subregions.

The first subregion overlapped the gene olfactory receptor family 2 subfamily T member 1 (*OR2T1*). There are various haplotypes, including three major haplotypes that we denote as types 1, 2, and 3, of which the type-1 haplotype was the most abundant (70.8%). Interestingly, the African contigs contained no type-2 or type-3 haplotypes (Figure 2C). The second subregion contained the gene olfactory receptor family 2 subfamily G member 6 (*OR2G6*). Although hg38 was composed of a segment pattern to which we refer as

medium-medium-medium (Figure 2A; subregion 2) for the middle three MA-blocks at this region, the contigs from various human populations actually reflected three segment patterns: long-medium, medium-long, and medium-short-medium. The type-1 haplotype (59.0%) was more dominant than the type-2 (25.0%) and type-3 (16.0%) haplotypes (Figure 2D). The third subregion spanned four genes (*OR2T34*, *OR2T10*, *OR2T11*, and *OR2T35*). The type-1 and type-3 haplotypes differed by only a single label. Despite this minor difference, only the contigs from the African and American populations had the type-3 haplotype. The type-2 haplotype was a deletion from the other types. The African contigs had a greater abundance (40.7%) of the type-2 haplotype than all other populations (15.7%) (Figure 2E). We also observed relationships in the haplotype distribution across two subregions. For example, type-2 haplotypes in subregion 3 are more likely to be followed by the type-1 haplotype (30.5%) than by the type-2 (3.1%) and type-3 (9.5%) haplotypes in subregion 2.

Copy number variations

OMMA not only allowed direct visualization of the presence of copy number variations (CNVs); it also enabled the deduction of the exact copy number of the query. In the second complex case, we illustrated the characterization of CNV based on multiple alignment of contigs spanning the gene *ANKRD30A*, which contained tandem repeats including a very large repeat unit of size 11.1 kb [11]. The multiple alignment of contigs containing the CNV easily revealed the variable length of the contigs within this region, in comparison to adjacent conserved regions that had mostly the same length in the various contigs (Figure 3A).

By choosing two flanking MA-blocks that corresponded to the boundary of the CNV region, the number of segments between them was used to deduce the number of copies in each contig. As an example, a contig from the sample NA19795 from the American population contains 16 alternating long and short segments. Because each repeat unit contains two segments (one long and one short), we deduced that this contig has eight copies. In comparison, the reference genome hg38 contains eight segments, or four copies. The number of tandem repeat copies in other contigs ranged from two to ten. Based on the copy numbers in different contigs of each population, we investigated the correlation between copy number and ethnicity and found that European contigs had fewer copies than other populations (with marginal statistical significance; Tukey test, $p = 0.06$) (Figure 3B).

Reconstruction of patterns in genomic regions not found in the reference

In the third case, we used multiple alignment to characterize the haplotype differences in the subtelomeric region of chromosome 20p, the sequence of which does not exist in the reference human genome. This region has been explored using optical maps and has been shown to display a pattern that is not found in the reference genome [12]. In our analysis, the extension of multiple alignment of the contigs beyond the sequence-containing portion of the reference chromosome 20p allowed us to discover a large indel (type-1: without inserted pattern; type-2: with inserted pattern) as a major haplotype difference among the contigs (Figure 4A). The reliability of this extension is confirmed by alignment of molecules (Figure S5). Notably, the African contigs contained only the type-1 haplotype, whereas the contigs from other populations contained both haplotypes (Figure 4B).

OMMA revealed conservation of genomic structures and predicted colicin and bacteriophage integration

We applied OMMA to optical maps generated from 21 drug-resistant *A. baumannii* genomes of various strains (Figure 5). Briefly, the optical mapping data were generated and assembled into a single consensus optical mapping assembly for each of the 21 samples. OMMA combined the 8315 segments from all strains into 823 MA-blocks.

The different regions in the multiple alignment could be roughly divided into three categories: (1) conserved among most strains, (2) conserved among close strains only, and (3) highly variable and not conserved across strains (Figure 5). Although the segments shared by most strains likely represented the evolutionarily conserved regions, the segments shared only among the close strains could be the key to separation among the clusters of strains. The highly variable segments, in contrast, could be hot spots for the integration of genomic islands.

The categories could be partially supported by different conservation levels of the MA-blocks. Most MA-blocks were conserved, and 23.8% of MA-blocks contained segments from all genomes, which accounted for 49.5% of all segments (Figure S6). These mainly constitute the region in category 1. Notably, some MA-blocks were evolutionarily conserved only within a certain set of genomes, as shown by the major peaks of

MA-blocks with 8 (12.1%) and 13 (12.8%) segments, that constitute the region in category 2. This local conservation is likely the result of our experimental strains that were distributed into two main groups with 8 and 13 strains, respectively. (See the phylogenetic tree analysis section below.) The remaining MA-blocks that are not conserved constitute the region in category 3.

Segments that were not completely conserved that involved size changes were of greater interest than those with only label additions or deletions because the former corresponded to the indel of a large piece of DNA, which would be more likely associated with the gain or loss of an entire set of functional elements, whereas the latter would be associated with the introduction or disruption of a single functional element due to single nucleotide variations or small indels. We deduced the identity of these functional elements by combining sequencing and optical mapping data (Figure 5). For example, a potential insertion was likely the integration of a 9286 bp sequence, including a Colicin V secretion gene *cvaA*. Another example was the integration of a 40,243 bp sequence, including several bacteriophage genes.

OMMA-based phylogenetic tree reconstruction revealed an evolutionary relationship among strains

Next, we applied our phylogenetic tree reconstruction method to reconstruct the phylogenetic tree of the 21 strains of *A. baumannii* based on their OMMA alignment (Figure 5). The reconstructed tree was separated into two major clusters of 13 and 8 strains. The larger cluster was further separated into two subclusters of nine and four strains, in line with their respective multilocus sequence typing (MLST) information. Specifically, the tree from optical mapping separated the samples into three clusters of sequence types (STs): (1) [ST75, ST92, ST137, ST346], (2) [ST254], and (3) [ST96]. In fact, the four STs in the first cluster were highly similar and differed only in the single nucleotide polymorphisms of a single gene.

One advantage of our method is that it separates strains based on whole-genome structures, unlike the traditional MLST method, which relies on only a selected set of genes. As a result, our method provides substantially greater detail about the evolutionary relationships among the strains. For example, the strains classified as ST96 by MLST could be further divided into multiple groups based on our results.

Discussion

Our OMMA method combines multiple queries into a single comparison, which assists with a wide range of analyses, including those in comparative genomics and population genomics. Multiple alignment provides a comprehensive view of the comparison of queries. With variations captured across different queries, our method is able to reflect the level of variability or conservation within a certain region and thus locate potential hotspots for genomic variations.

OMMA is also a useful tool for population genomics analyses. Variable haplotypes can be classified quickly, and their relative abundance in various populations can then be directly visualized and analyzed. With the phylogenetic tree reconstructed, the elements responsible for the differentiation into clusters can also be determined.

One major advantage of the use of optical mapping over sequencing is its ability to directly visualize and study the genome structure. The assembled optical mapping contigs are usually much longer than sequence assemblies that usually break at short repetitive regions. It also becomes more challenging when we need to characterize complex structural changes in larger genomes like the human genome by examining sequence assembly.

The OMMA pipeline offers great flexibility in customizing procedures. The flexibility of this method allows the task of multiple alignment of a very large genome to be divided into smaller jobs. The results can then be combined into a single multiple alignment during the final step. Our method also supports aligning queries with rearrangements including inversions and intrachromosomal translocations. In addition to the applications demonstrated here, our multiple alignment method could be extended for other studies, such as pan-genome construction and genomic island prediction. Our method has limitations, including a low tolerance to very large segmental duplications (e.g., 250 kbp) with multiple copies. However, with the recently launched DLE-1 labeling chemistry that no longer introduces double-strand DNA breaks at two closely-located nicks, the improved quality of assemblies should reduce the effect of this limitation on multiple alignment.

Conclusions

In this paper, we describe the first rearrangement-tolerant and nonproprietary multiple alignment method for optical mapping of data. The method's accuracy was assessed using an *A. baumannii* dataset with high

precision and recall. We demonstrate the application of the multiple alignment results to phylogenetic analyses and complex region analyses (e.g., multiple haplotypes, CNVs, and novel genomic region). OMMA could serve as a fundamental tool for the further development of more specific analytic methods for the study of comparative and population genomics.

Methods

OMMA overview

The OMMA program was developed to compute segment-matching information to generate a series of MA-blocks as a collection of matching segments of entries. The entire pipeline is separated into a preparation step and a multiple alignment step that can be further divided into three main substeps (modules) (Figure 1). In the first substep, the matching segments from pairwise alignments or other sources are used to construct the blocks. In the second substep, these blocks are sorted to minimize rearrangement events. Finally, in the third substep, proximal segments are merged if their sizes are similar.

Preparation step

At the preparation step before the core modules are run in multiple alignment, we must generate some clues about which segments on different queries should be put together. Usually, this process considers not only the size matching of a pair of target segments alone but also accounts for the size matching of their proximate segments. We define a piece of evidence that helps to determine the matching of segments as a *source*. A source is composed of a set of *segment links* that are denoted as a matching pair of segments from different queries. Our method accepts two sources that provide clues about the segment links based on similar labeling patterns: pairwise alignment among queries and the multiple alignment results.

Pairwise alignment result as sources

All queries are aligned in a pairwise manner (Figure 1B), followed by derivation of the segment links from the alignments of each pair of queries. The pairwise alignment results were generated by OMBlast [5], which could output partial alignments (local alignments between a pair of queries) that are critical to multiple alignment of regions with rearrangement.

Multiple alignment result as a source

The multiple alignment result (generated by the OMMA pipeline) can also be used as a source because the results are intuitively a collection of matching segments. The result is particularly useful in large-scale multiple alignment, such as whole-genome multiple alignment in humans, for which one-step multiple alignment is not computationally feasible. By dividing the large number of queries into separate subtasks, several multiple alignments on a smaller scale can be achieved, and the results can serve as the sources for a global multiple alignment.

MA-block construction

Construction of MA-blocks based on segment links

For multiple alignment of Q queries, each MA-block is represented by a binary vector of length Q, where a "non-empty" entry or "1" indicates that a query participates in this block, and an "empty" entry or "0" indicates that it does not. A non-empty entry can be occupied by only one segment from the query. No more than one segment from the same query can be assigned to each MA-block.

An undirected graph is constructed with the segments as vertices and the segment links as edges (Figure 1C). Each connected component is treated as an MA-block *candidate*. A *valid* MA-block is defined as a collection of segments with no more than one segment from the same query. If an MA-block candidate fulfills this criterion, all segments in the candidate are directly converted into an MA-block. Otherwise, each individual segment in the candidate is instead assigned to a separate MA-block.

Segment links from multiple sources

The selection of a proper set of parameters in pairwise alignment is difficult. To solve this problem, segment links from various sources can be supplied in the multiple alignment processes. Briefly, segment links from the most confident sources (such as pairwise alignment results with more stringent parameters) are first used to construct MA-blocks. The segment links from less confident sources (such as pairwise alignment results with more lenient parameters) are then used to connect the MA-blocks. Two connected MA-blocks are merged if their binary vector does not overlap (i.e., if they do not have segments from the same queries after merging).

MA-block sorting

The MA-blocks are sorted to better illustrate the overall pattern of the queries. Here, we briefly demonstrate the idea using Figure 1D before discussing the details of implementation. With different MA-blocks generated from the previous step, it is obvious to place MA-block [A1, B1, C1] before MA-block [A2, B2, C2] (i.e., segment A1 is followed by segment A2 on query A, and the same applied to query B and C), because the segments from all queries in these two blocks are consecutive. However, the problem becomes more complicated after rearrangement such as inversion or translocation. For example, we must choose what follows the MA-block [A3, B3, C3]; it could be MA-block [A4, C8] (i.e., because segment A3 is followed by segment A4 on query A), MA-block [B4] (i.e., because segment B3 is followed by segment B4 on query B), or MA-block [A8, B9, C4] (i.e., because segment C3 is followed by segment C4 on query C).

More formally, the MA-blocks are sorted to minimize the number of rearrangement events and maximize the matching events. For B MA-blocks constructed from the previous module, consider a nonempty qth entry in the bth MA-block and a nonempty qth entry in the $(b+x)$th MA-block, where x is a positive integer and any qth entries are empty in the $(b+1)$th to $(b+x-1)$th MA-blocks. A *matching event* occurs if the two entries represent consecutive segments in the original qth query and have the same orientation. In contrast, a *rearrangement event* occurs when the two entries are in different orientations or if they are not consecutive segments ordered in the original qth query. Note that the sum of matching and rearrangement events in the final chain remains constant for the same set of queries. This problem is equivalent to a nondeterministic polynomial-time hard (NP-hard) traveling salesman problem, in which all vertices (MA-blocks) must be

traversed exactly once, except that no limitation is set on the start and end points. Because determination of an optimized solution is computationally intensive, the nearest neighbor-joining algorithm was devised to approximate a suboptimal solution.

Nearest neighbor–joining algorithm

In the nearest neighbor-joining algorithm, the MA-blocks are connected into multiple chains. The goal is to repeatedly connect chains until a single chain remains as the solution to the order of the MA-blocks. In the beginning, one chain is created for each MA-block, resulting in C chains. The connection candidates from each pair of chains are added into a priority queue. In each round of connection, a pair of chains that results in the highest connection priority is polled from the queue and connected to a new chain, followed by an update on the connection candidates for the new chain. The connection process is repeated for $C-1$ rounds to connect all C chains into one single chain.

Connection priority for two chains of MA-blocks

The connection priority for two chains of MA-blocks is based entirely on the relationship of the entries in the two chains. In total, Q relationships are built based on Q sets of entries compared within the same query. Only the last nonempty entry from the former chain and first nonempty entry from latter chain are compared. We define the relationship of the entries from a particular query as matching and rearrangement if the two selected entries are consecutive and nonconsecutive segments, respectively. In addition to matching, if the two entries are taken from the last MA-block of the former chain and first MA-block of the latter chain, they are further categorized as direct matching. The relationship is set as empty if no nonempty entry exists in either chain. An example is shown in Figure S8, in which the relationships of the four sets of entries from queries A, B, C, and D represent matching, direct matching, rearrangement, and empty relationships, respectively. The parameters are defined for the count of relationships between the two chains as follows:

- n_m: matching (note that a direct matching relationship is also counted here)
- n_d: direct matching
- n_r: rearrangement

- n_{rr}: reference rearrangement (if reference is provided by the user)
- n_e: empty

The parameters above always meet the following criteria:

- $n_m+n_r+n_e=Q$
- $n_d \leq n_m$.
- $n_{rr} \leq n_r$.

The connection priority for a pair of chains is listed below:

1. At least one matching relationship exists ($n_m \geq 1$)
2. No rearrangement is found ($n_r=0$)
3. If no rearrangement is found, n_d is maximized, followed by maximizing n_m
4. If rearrangement is found,
   (a) the connection results in no rearrangement in reference ($n_{rr}=0$, if reference is provided by the user)
   (b) n_r is minimized, followed by maximizing n_m, followed by maximizing n_d

Directed graph representation for multiple alignment

The multiple alignment results can be represented as an acyclic directed graph. Consider an acyclic directed graph constructed with MA-blocks as the vertices. A directed edge from MA-block b to $b+x$ is built if two nonempty entries exist from the same query q in bth and $(b+x)$th MA-blocks, where x is a positive integer and all entries from query q are empty in the $(b+1)$th to $(b+x-1)$th MA-blocks. An edge is built between two MA-blocks with a weight equal to the sum of the matching and rearrangement relationships. Its direction follows the order of the MA-blocks. Such a representation simplifies the interpretation of the actual variation and noise, which can be removed by filtering the edges with minimum weight.

Merging

We wish to merge MA-blocks without introducing new rearrangements. The order of the MA-blocks other than the merging target remains unchanged in the merging actions. With such a constraint, the merging step can effectively increase the sensitivity of the final multiple alignment result. From the directed graph representation of the sorted MA-blocks, the merging of two MA-blocks leads to new rearrangements if the set of descendant vertices of one MA-block intersects the set of ancestor vertices of another MA-block.

Merging of MA-blocks by proximity

Consider a directed graph representation of the sorted MA-blocks (Figure 1E), with MA-blocks as vertices. Two MA-blocks that share at least one incoming neighbor or one outgoing neighbor are defined as proximate. Two proximate MA-blocks are merged if the average size of the segments in one MA-block matches those in the other MA-block. The merging of two MA-blocks can be disruptive and lead to rearrangement. To avoid the introduction of new rearrangements, we must check for overlapping of the ancestor and descendant vertices. The ancestor and descendant vertices of an MA-block are obtained by traversing the directed graph, and the ancestor and descendant vertices are updated by dynamic programming. After each merging step, the directed graph and the ancestor and descendant vertices are updated.

Merging of MA-blocks by segment links

Another merging strategy relies on segment links, as described above in the MA-block construction step. This time, the connections are taken as evidence for the potential merging of two MA-blocks that include two segments that form a connection. Like merging proximate MA-blocks, the checking and update steps described in the previous section are used to prevent the introduction of rearrangements during the merging of two connected MA-blocks.

Assessment of accuracy of OMMA

Because there is no standard answer for multiple alignment based merely on optical mapping patterns, the accuracy of the current method was inferred from the consistency attained among multiple alignments from optical mapping and multiple alignments from sequencing. Multiple alignment software based on genomic

sequences was used to assess the accuracy of multiple alignment in optical mapping. In this study, the genomic sequences were aligned using Mugsy v1r2.3 [13] which used results from MUMmer 3.20 [14] under the default parameters.

The accuracy was measured by two parameters, precision and recall. In multiple alignment of optical mapping, a segment *i* forms segment-pair $p_{i,j}$ with another segment *j* for $i \neq j$ if the two segments belong to the same MA-block. In multiple alignment from sequencing, consider a segment *m* of length l_m, a segment *n* of length l_n, and the length of multiply aligned sequence l_{seq}. The similarity $s_{m,n}$ of segment *m* to segment *n* is defined as $\frac{l_{seq}}{l_m}$. The segment *m* forms segment-pair $p_{m,n}$ with segment *n* if:

$$s_{m,n} \geq 0.8 \text{ and } 0.8 \leq \frac{l_m}{l_n} \leq 1.25$$

An intersected segment-pair set was created by the intersection of a segment-pair set derived from the multiple alignment of optical mapping and one derived from multiple alignment of sequence. Precision was calculated as the number of intersected segment-pairs divided by the number of segment-pairs derived from the multiple alignment of optical mapping. Recall was calculated as the number of intersected segment-pairs divided by the number of segment-pairs derived from the multiple alignment of sequence.

Phylogenetic tree reconstruction

Our reconstruction method is based mainly on the assumption that two strains have greater similarity if they share more MA-blocks in the multiple alignment. From the multiple alignment, the distance of sample *i* relative to sample *j* is defined as:

$$d_{ij}=1-\frac{\frac{f_{ij}}{f_{jj}}+\frac{f_{ji}}{f_{ii}}}{2}$$

where f_{ij} represents the number of MA-blocks shared by *i* and *j*. Note that $f_{ii} \geq f_{ij}$, $f_{ij}=f_{ji}$, and $d_{ij}=d_{ji}$.

A distance matrix was built to reconstruct the phylogenetic tree via an unweighted pair-group method with an arithmetic mean approach. The tree was then visualized with the "APE" package in R [15].

Assessment of phylogenetic tree accuracy

The performance of our method of phylogenetic analysis was assessed using simulated genomes. The virtual genomes were simulated according to a virtual phylogeny in Figure S9. Briefly, in the first generation, random mutations were introduced into an ancestor genome to simulate two children genomes. These genomes were then taken as the parent genomes in the next generation to synthesize more children genomes. At the *n*th generation, 2^n children genomes were simulated. The children genomes at the last generation were used for the assessment of accuracy. The mutations were generated based on the pIRS program [16].

Generation of optical mapping and sequencing data

Bacteria sample collection

All 21 isolates were sampled from inpatient specimens or colonization studies in children for *A. baumannii*. The isolates selected for this project had been independently characterized by pulsed-field gel electrophoresis and/or MLST. The results were unknown to the optical mapping analysis procedure.

Genomic DNA extraction

Megabase-sized bacterial genomic DNA was extracted with the agarose gel plug method. Embedment in the porous agarose matrix protected the DNA from physical shearing while allowing access of restriction enzymes. The bacterial cell pellets were resuspended and embedded in low-melting agarose to form gel plugs. The plugs were treated with proteinase K and lysozyme for cell lysis. After washing several times in 1X TE buffer, the plugs were melted at 70°C for 2 min and equilibrated at 42°C for 5 min before Gelase (Epicentre) was added to solubilize the sample. The solubilized DNA obtained was concentrated by drop-dialysis with 1X TE buffer for 2.5 h at room temperature. The high–molecular weight DNA samples were then quantified with a Quant-iTdsDNA Assay Kit (Invitrogen/Molecular Probes). The DNA quality was checked by contour-clamped homogenous electric field gel electrophoresis.

Nicking, labeling, and repairing reactions

The bacterial DNA samples were nicked, labeled, repaired, and stained. In summary, the single-strand breaks (nicks) were introduced to 300 ng bacterial DNA by nicking endonuclease Nt.BspQI (New England Biolabs) at 37°C for 2 h. The DNA nicks were filled with fluorescent nucleotides by Taq polymerase and sealed with Taq DNA ligase (New England Biolabs). The backbone of the double-stranded DNA was stained overnight with fluorescence dye YOYO-1 (Invitrogen).

Imaging and raw data processing

The stained double-stranded DNA with fluorescent labels was loaded by electric current onto a chip that contained massively parallel nanochannel arrays, upon which the DNA was linearized and imaged. The lengths and relative positions of the fluorescent labels of the DNA molecules were calculated from the images to individual single-molecule maps (optical maps) by estimating any errors in size scaling and missing or spurious labels.

Optical map assembly

The optical mapping assembly of the genome of *A. baumannii* was performed using the standard pipeline in "Bionano Solve 3.1" [7], followed by a custom refinement script to trim the contigs. The plasmids and incomplete genomes were removed from further analysis. Because bacterial genomes are circular, for ease of analysis and visualization, all genomes were oriented such that they all began with a conserved pattern across most *A. baumannii* strains.

Sequencing data generation and sequence assembly

To reveal the identity of the variations detected by optical mapping, six *A. baumannii* samples were selected for sequencing and assembled using SOAPdenovo [17]. The assembled contigs were annotated using Prokka [18]. To deduce annotations on optical mapping contigs, the assembled sequence contigs were aligned on the optical mapping contigs using OMBlast [5], with annotations assigned on the relative aligned position on the optical mapping contigs.

Human optical maps data for validation

The generation of assembled contigs of 154 human individuals from five super-populations (AFR, AMR, EAS, EUR, SAS) used for characterization of complex genomic variations was reported by Levi-Sakin et al. (manuscript under review).

Availability of supporting source code and requirements

Project name: OMTools Project

Project home page: https://github.com/TF-Chan-Lab/OMTools

Operating system: Platform independent

Programming language: Java

Other requirements: Java 8 or higher

License: GNU GPL

The OMMA is a new module in the OMTools package as published in [19].

Availability of supporting data

The raw optcal mapping and sequencing data of the *A. baumannii* strains are available at NCBI (Accession ID pending).

Declarations

Ethics approval and consent to participate

Not applicable

Consent for publication

Not applicable

Competing interests

The authors declare that they have no competing interests. The technology platform described in this paper was developed by BioNano Genomics (San Diego, CA).

Funding

TFC, KYY, and PLH are partially supported by a Health and Medical Research Fund (HMRF12110542) from the Food and Health Bureau of the Hong Kong Special Administrative Region (HKSAR). AKYL and TFC are partially supported by a General Research Fund (14102014), Collaborative Research Fund (C4042-14G), an Area of Excellence Scheme (AoE/M-403/16) from the HKSAR Research Grants Council, and a funding from the Innovation and Technology Commission, Hong Kong Government to the Partner State Key Laboratory.

Author's contributions

TFC and KYY conceived the study. AKL, TFC, and KYY designed the computational methods. AKL implemented the computational methods. PYL and PLH provided the DNA from *A. baumannii* samples and conducted the MLST and PFGE experiment. YYL, CC, and PYK produced the optical mapping data of *A. baumannii*. AKL processed the optical mapping data. AKL, TFC, and KYY wrote the manuscript. All authors read and approved the final manuscript.

References

1. Dimalanta ET, Lim A, Runnheim R, Lamers C, Churas C, Forrest DK, et al. A microfluidic system for large DNA molecule arrays. Analytical chemistry. 2004;76:5293–5301.

2. Lukinavičius G, Lapiene V, Staševskij Z, Dalhoff C, Weinhold E, Klimašauskas S. Targeted labeling of DNA by methyltransferase-directed transfer of activated groups (mTAG). Journal of the American Chemical Society. 2007;129:2758–2759.

3. Grunwald A, Sharim H, Gabrieli T, Michaeli Y, Torchinsky D, Juhasz M, et al. Reduced representation optical methylation mapping (R2OM2). bioRxiv. 2017;113522.

4. McCaffrey J, Sibert J, Zhang B, Zhang Y, Hu W, Riethman H, et al. CRISPR-CAS9 D10A nickase target-specific fluorescent labeling of double strand DNA for whole genome mapping and structural variation analysis. Nucleic acids research. 2015;44:e11–e11.

5. Leung AK, Kwok TP, Wan R, Xiao M, Kwok PY, Yip KY, Chan TF. OMBlast: alignment tool for optical mapping using a seed-and-extend approach. Bioinformatics. 2017;33:311–319.

6. Mendelowitz LM, Schwartz DC, Pop M. Maligner: a fast ordered restriction map aligner. Bioinformatics. 2016;32:1016-1022

7. Shelton JM, Coleman MC, Herndon N, Lu N, Lam ET, Anantharaman T, et al. Tools and pipelines for BioNano data: molecule assembly pipeline and FASTA super scaffolding tool. BMC genomics. 2015;16:734.

8. Valouev A, Li L, Liu YC, Schwartz DC, Yang Y, Zhang Y, Waterman MS. Alignment of optical maps. Journal of Computational Biology. 2006;13:442-462.

9. http://www.applied-maths.com/bionumerics. Accessed 15 Jul 2018.

10. Young JM, Endicott RM, Parghi SS, Walker M, Kidd JM, Trask BJ. Extensive copy-number variation of the human olfactory receptor gene family. The American Journal of Human Genetics. 2008;83:228–242.

11. Warburton PE, Hasson D, Guillem F, Lescale C, Jin X, Abrusan G. Analysis of the largest tandemly repeated DNA families in the human genome. BMC genomics. 2008;9:533.

12. McCaffrey J, Young E, Lassahn K, Sibert J, Pastor S, Riethman H, et al. High-throughput single-molecule telomere characterization. Genome research. 2017;27:1904–1915.

13. Angiuoli SV, Salzberg SL. Mugsy: fast multiple alignment of closely related whole genomes. Bioinformatics. 2011;27:334–342.

14. Kurtz S, Phillippy A, Delcher AL, Smoot M, Shumway M, Antonescu C, et al. Versatile and open software for comparing large genomes. Genome biology. 2004;5:R12.

15. Paradis E, Claude J, Strimmer K. APE: analyses of phylogenetics and evolution in R language. Bioinformatics. 2004;20:289–290.

16. Hu X, Yuan J, Shi Y, Lu J, Liu B, Li Z, et al. pIRS: Profile-based Illumina pair-end reads simulator. Bioinformatics. 2012;28:1533–1535.

17. Luo R, Liu B, Xie Y, Li Z, Huang W, Yuan J, et al. SOAPdenovo2: an empirically improved memory-efficient short-read de novo assembler. Gigascience. 2012;1:18.

18. Seemann T. Prokka: rapid prokaryotic genome annotation. Bioinformatics. 2014;30:2068–2069.

19. Leung AK, Jin N, Yip KY, Chan TF. OMTools: a software package for visualizing and processing optical mapping data. Bioinformatics. 2017;33:2933–2935.

Main figures

Figure 1: Overview of the OMMA pipeline. Details of these algorithms are described in Methods. (A) Example queries for multiple alignment. (B) Pairwise alignments of the queries to create sources for segment links. An insertion/deletion event occurs between queries A and B (see segments A4, B4, and B5). An inversion event occurs between queries A and C (see segments A4-A8 and C4-C8). (C) MA-block construction. An undirected graph is constructed with segments as vertices and segment links as edges. An MA-block candidate (connected component in the undirected graph) that fulfills the MA-block criteria (e.g., [A1, B1, C1]) is converted directly into an MA-block [A1, B1, C1]. In contrast, segments in an MA-block candidate that does not fulfill the criteria (e.g. [A5, A6, B6, B7, C6, C7]) are broken into individual MA-blocks [A5], [A6], [B6], [B7], [C6], and [C7]. (D) MA-block sorting. Matching events between two MA-blocks (dotted arrow) are determined by any proximate segment (e.g., A1 to A2 or C3 to C4). Red dotted arrows highlight matching events within the inverted region of query C. Their suggested direction is opposite that from queries A and B. The MA-blocks are sorted to minimize rearrangement events. Here two

rearrangement events occur when joining segments C3-C8 (MA-blocks 3 and 4) and C4-C9 (MA-blocks 14 and 15). The sorted MA-blocks are packed for purposes of visualization, with black dotted lines indicating empty space, and red dotted circles indicating rearrangement events. (E) MA-block merging. Without contradicting the order of MA-blocks determined in the last module, MA-blocks that share similar segment sizes are merged. MA-blocks [A6], [B7], and [C6] share a similar size and are proximate to the MA-block [A7, B8, C5] (purple) and are merged into one MA-block. Similarly, MA-blocks [A5], [B6], and [C7] share a similar size and are proximate to the MA-block [A6, B7, C6] (yellow) and are merged into another MA-block.

Figure 2: Multiple indels at the olfactory receptor region (1q44). (A) Three subregions that overlapped olfactory receptor genes with multiple variations were characterized. The multiple alignment shows the patterns of hg38 and major haplotypes of other contigs for each subregion. Only American contigs are shown for illustration purposes. Each row represents a contig. The multiple alignment of the contigs from all populations is shown in Figure S2. (B) Alignment of optical maps from the American contigs on chromosome 1 was visualized using IrysView. The corresponding subregions shown in A are highlighted. It is noticeably difficult to resolve and characterize the labeling patterns in the presence of multiple haplotypes. (C-E) Contig representation at subregion 1-3. In subregion 1 (C), the African contigs only contained the type-1 haplotype. In subregion 2 (D), the contig ratio was similar among the various populations. In subregion 3 (E), only the African and American contigs had the type-3 haplotype. The African contigs also had more type-2 haplotypes than the other populations. Abbreviations for the super-population code are as follows: AFR, African; AMR, Ad Mixed American; EAS, East Asian; EUR, European; SAS, South Asian.

Figure 3: Copy number variation within the gene *ANKRD30A*. (A) Top: Example contig that contained the target CNV. Bottom: Multiple alignment of contigs from the American population offered a direct view of the whole CNV region. The number of copies in each contig was visualized directly, with each copy containing two labels. Only American contigs are shown for illustration purposes. The multiple alignment of contigs from all populations is shown in Figure S3. (B) CNVs at *ANKRD30A* across various populations. In general, the European contigs had fewer copy numbers.

Figure 4: Subtelomeric region 20p. (A) Multiple alignment of the contigs from the American population that extend beyond the human reference hg38. The haplotype difference was visualized in a region not covered by hg38. Only American contigs are shown here for illustration purposes. The multiple alignment of the contigs from all populations is shown in Figure S4. The labeling pattern from individual GM19921 beyond chromosome 20p was supported by pairwise alignment of optical maps (Figure S5). (B) Contig representation of the two haplotypes in different populations at subtelomeric region 20p. Contigs from the African population were devoid of the type-2 haplotype.

Figure 5: Overview of multiple alignment of 21 *A. baumannii* genomes. In the multiple alignment, the rectangles of various colors across a row represent segments from a query optical map. To indicate the alignment relationship of these segments, the segments in the same MA-block share a color and are arranged in the same column. Neighboring MA-blocks also share a color if they involve segments from the same set of optical maps. Black horizontal lines indicate empty spaces, and black circles indicate rearrangements in the multiple alignments. A portion of the multiple alignment is magnified to demonstrate the division of genome regions into (1) those conserved among most strains, (2) those conserved only among close strains, and (3) highly variable regions that are not conserved across strains. An example of the captured region indicates the integration of Colicin V secretion genes. Another example of bacteriophage gene integration is shown in Figure S7, which further describes the rearrangement event. The phylogenetic tree on the left was reconstructed based on the OMMA multiple alignment of the optical maps of the 21 *A. baumannii* strains. The strains were separated into two major clusters of 13 and 8 strains, and the larger cluster was further divided into clusters of 9 and 4 strains.

Figure 1

Click here to download Figure Figure1.ai

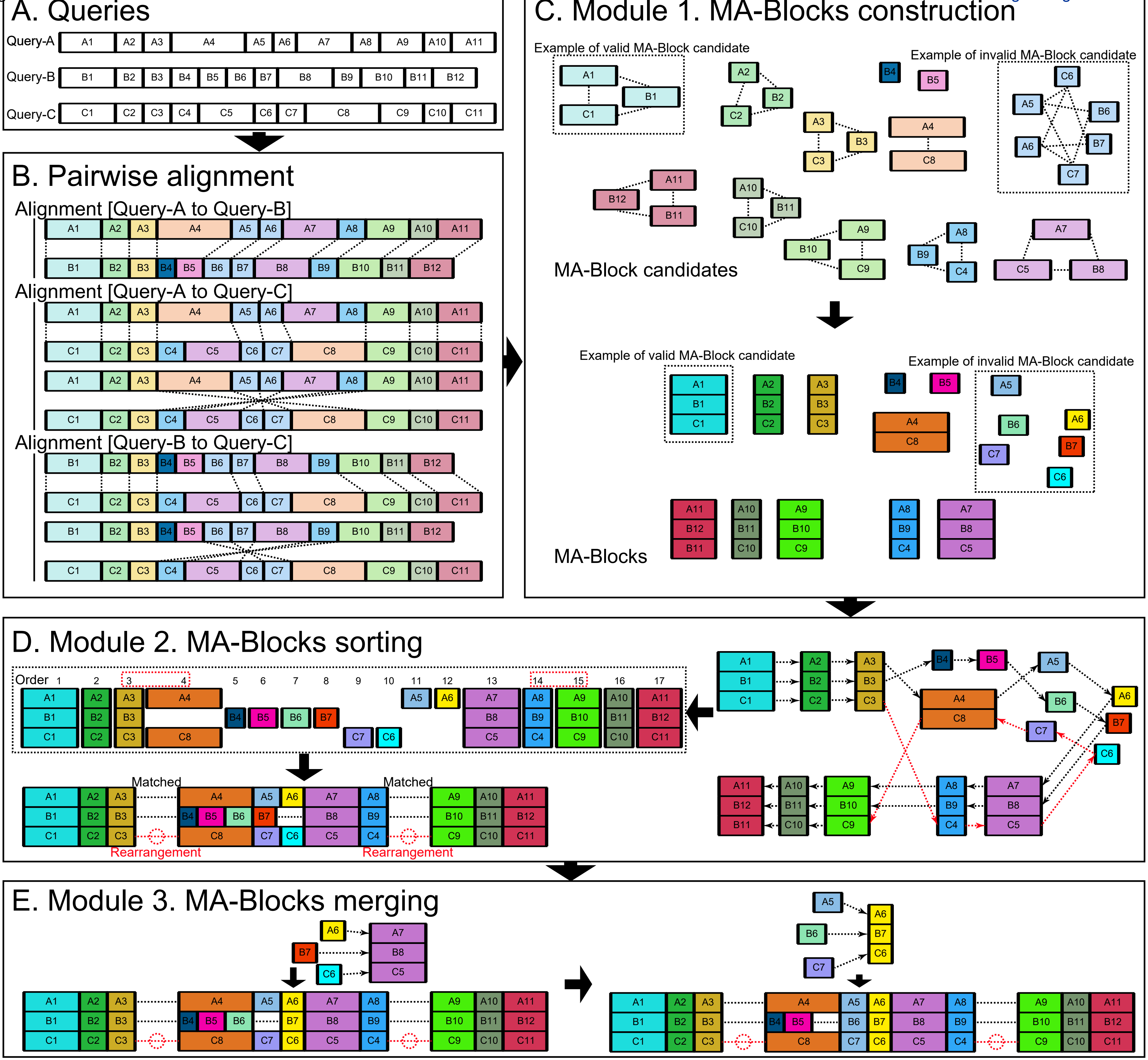

Figure 2

Click here to download Figure Figure2.ai

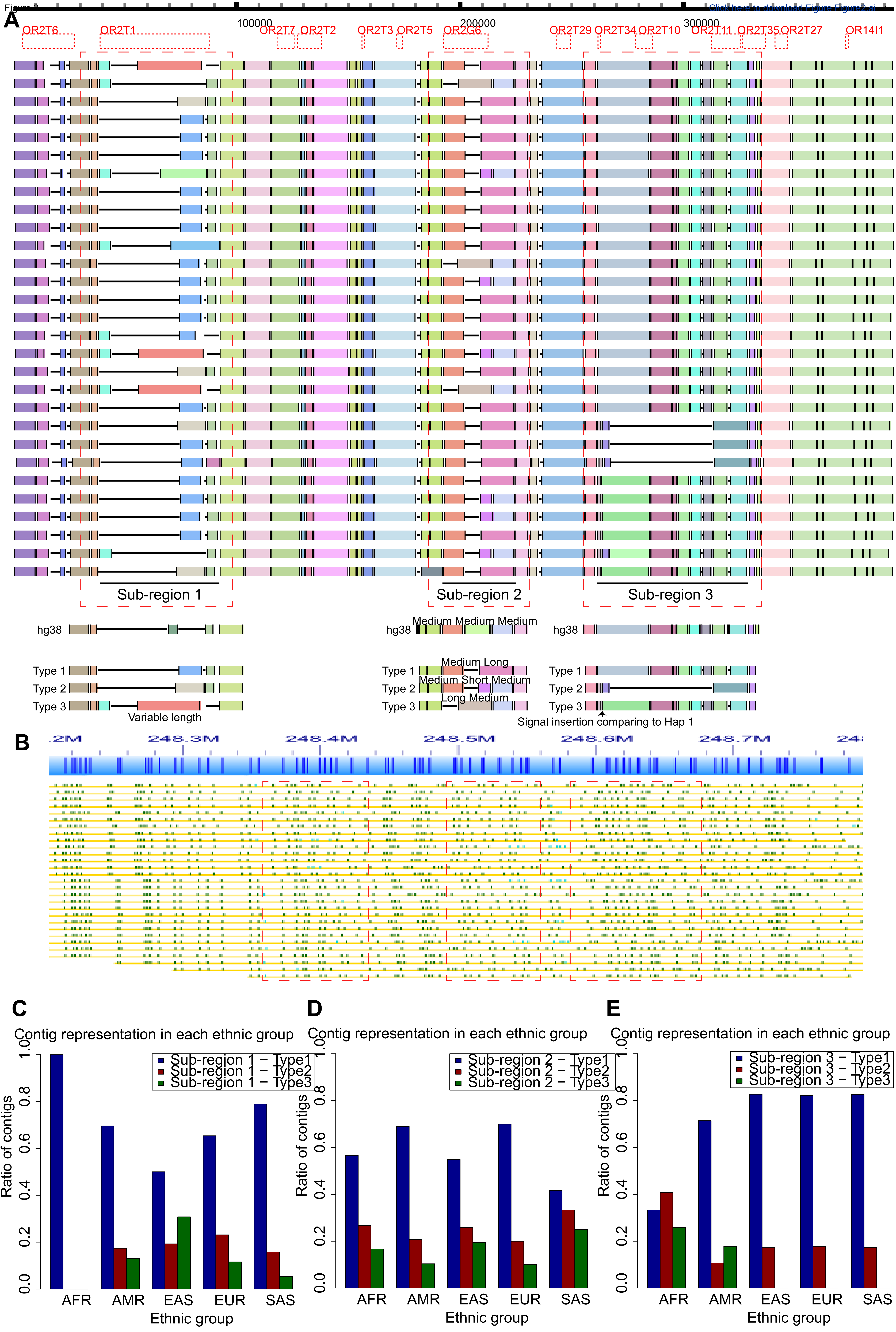

Figure 3

Click here to download Figure Figure3.ai

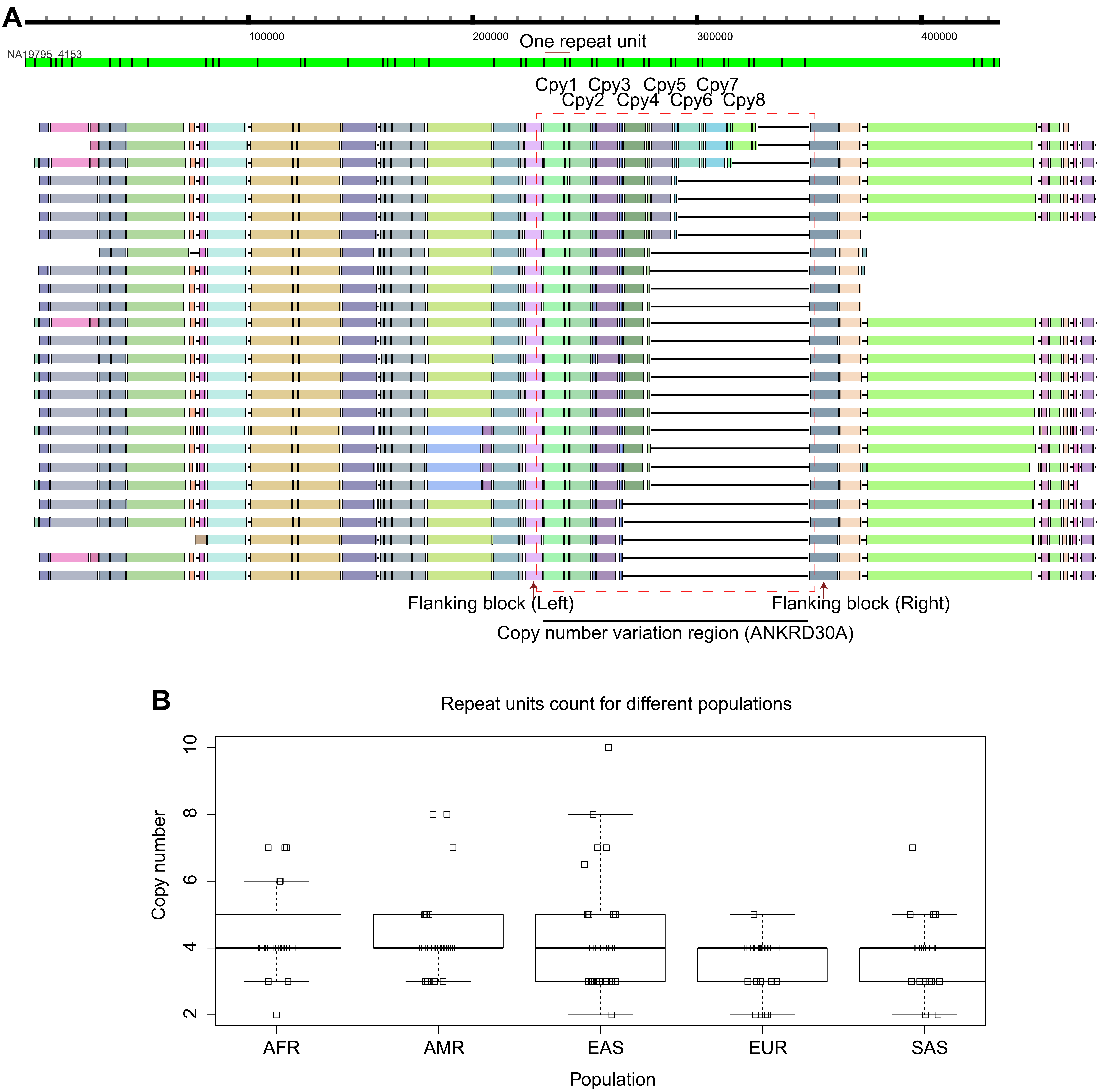

Click here to download Figure Figure4.ai

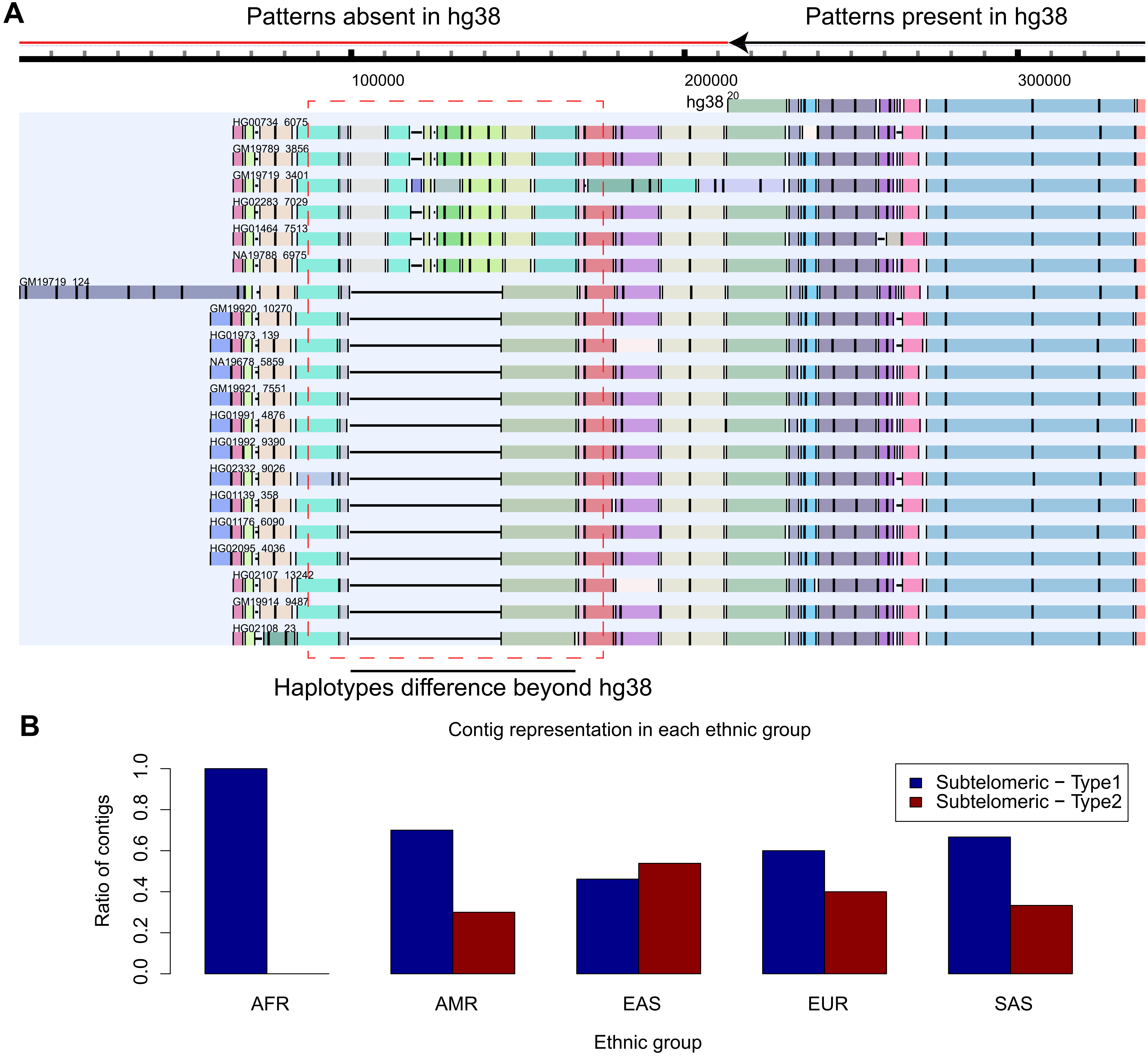

Figure 5

Click here to download Figure Figure5.ai

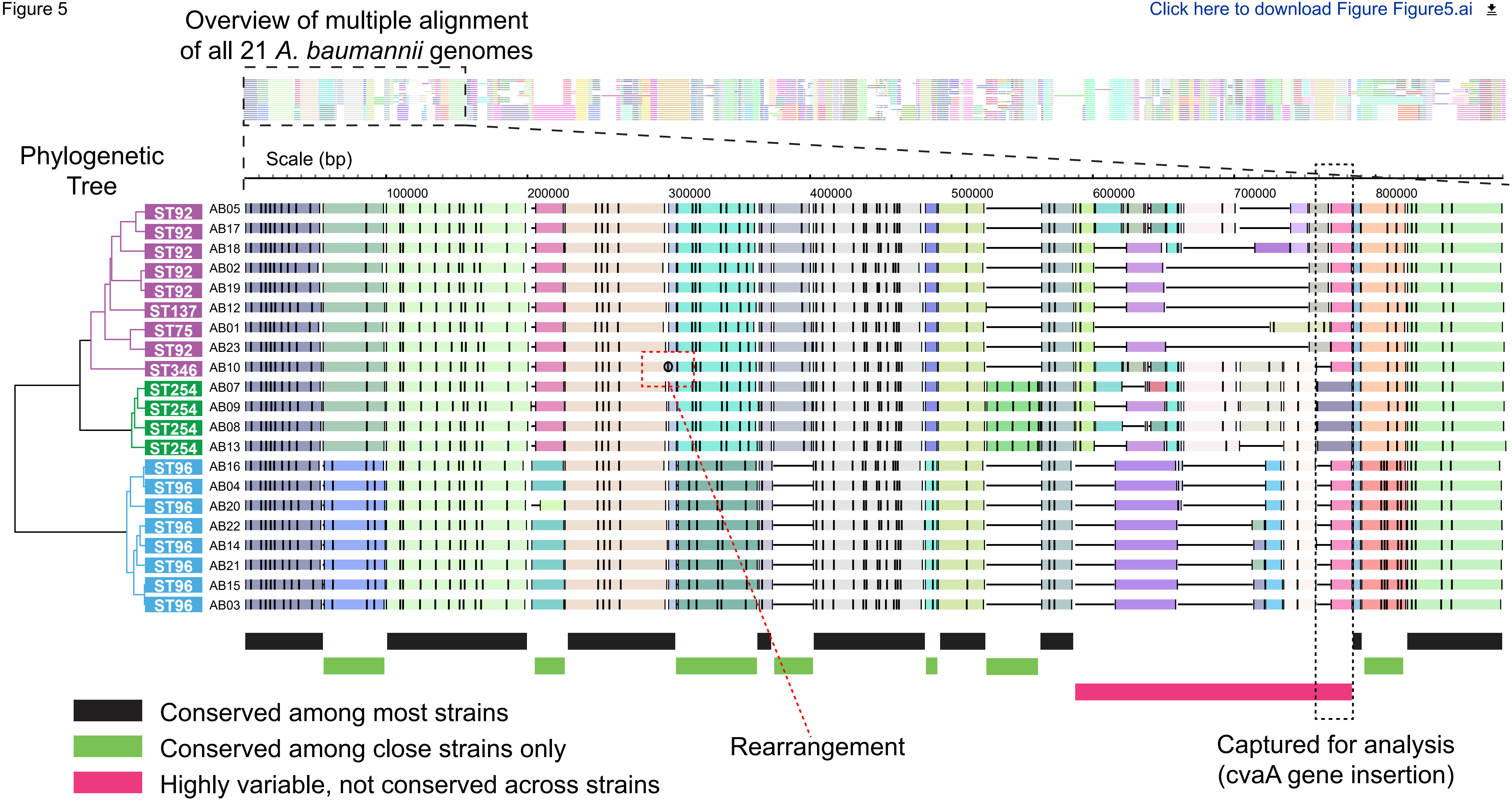

Click here to access/download
Supplementary Material
20180726_SupplementaryFigures.docx

Cover letter
Click here to download Personal Cover 20180803_CoverLetter.pdf

The Chinese University of Hong Kong
School of Life Sciences
香港中文大學
生命科學學院
香港中文大學五十周年
50th Anniversary of CUHK

CUHK, Shatin, NT, HK
香港新界沙田香港中文大學
TEL 電 話：(852) 3943 6122
FAX 圖文傳真：(852) 2603 5646
WEBSITE 網址：http://www.cuhk.edu.hk/lifesciences
E-MAIL 電郵：lifesciences@cuhk.edu.hk

August 5th, 2018

GigaScience
Oxford University Press

Dear Editors,

We would like to submit our manuscript, entitled "OMMA enables population-scale analysis of complex genomic features and phylogenomic relationships from nanochannel-based optical maps", for consideration for publication as a Technical Note in *GigaScience*.

Optical mapping, a single-molecule imaging technique that captures sequence-specific patterns along DNA molecules of several hundred kilobases in lengths, has become a complementary technology to sequencing-based methods in areas such as genome assembly and detection of large structural variation. We have previously published a series of algorithms for optical maps analysis. All of the currently available methods for optical mapping analysis take reference alignment as an essential step. This greatly restricts optical mapping to pairwise comparison. We present our method OMMA (optical mapping by multiple alignment), which allows simultaneous comparison among many samples without the need of a reference. When applying to a large experimental dataset of optical maps from 154 human sample across the 26 populations sequenced in the 1000 Genomes Project, OMMA could clearly resolve and characterize complex genomic features such as regions with multiple haplotypes, copy-number variations, and subtelomeric structures between ethnic groups. OMMA is one of the core methodologies adopted in an international collaborative study that is under final revision for *Nature Genetics*. When applying to small genomes such as bacteria, OMMA could reconstruct the phylogenomic relationships, and identify highly variable regions across different bacterial strains. These results were independently validated by conventional strain-typing and sequencing methods. With the increasing data throughput of optical mapping, the use of this technology in comparative genome analysis across many samples will soon become a reality. We believe OMMA a timely solution that can benefit the research community.

Thank you for your attention and we hope this work on OMMA can be considered for review.

Sincerely,

Ting-Fung Chan
Associate Professor
School of Life Sciences
State Key Laboratory of Agrobiotechnology
The Chinese University of Hong Kong
Hong Kong SAR
