# Supplementary material for: OMMA enables population-scale analysis of complex genomic features and phylogenomic relationships from nanochannel-based optical maps
Source: Gigascience. 2019 Jul 9;8(7):giz079. doi: 10.1093/gigascience/giz079 (PMC6615982; doi:10.1093/gigascience/giz079)
Supplement: giz079_Supplemental_File [file giz079_supplemental_file.docx]

## Supplementary figures


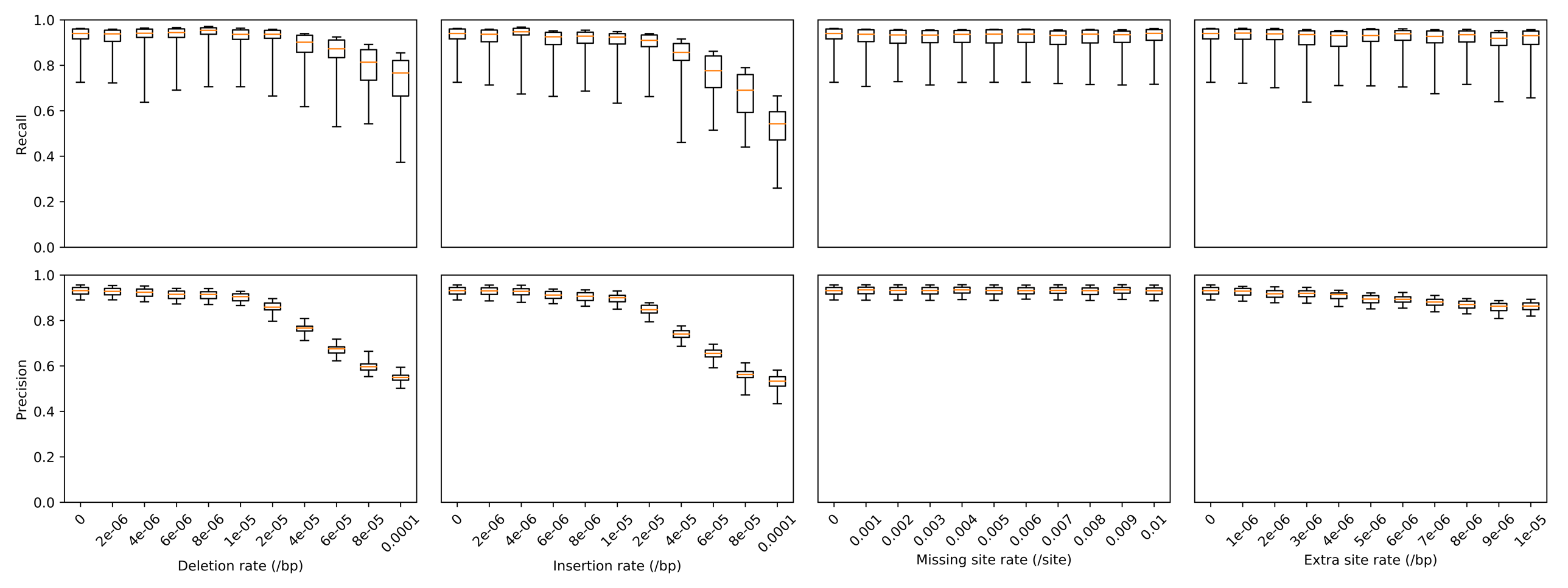


Figure S1. The performance of multiple alignment on genomes with various types of errors (missing site, extra site, segmental insertion and segmental deletion) at different rates. Each column of the figure shows the type of error introduced. The top and bottom rows show the recall and precision of multiple alignment respectively.


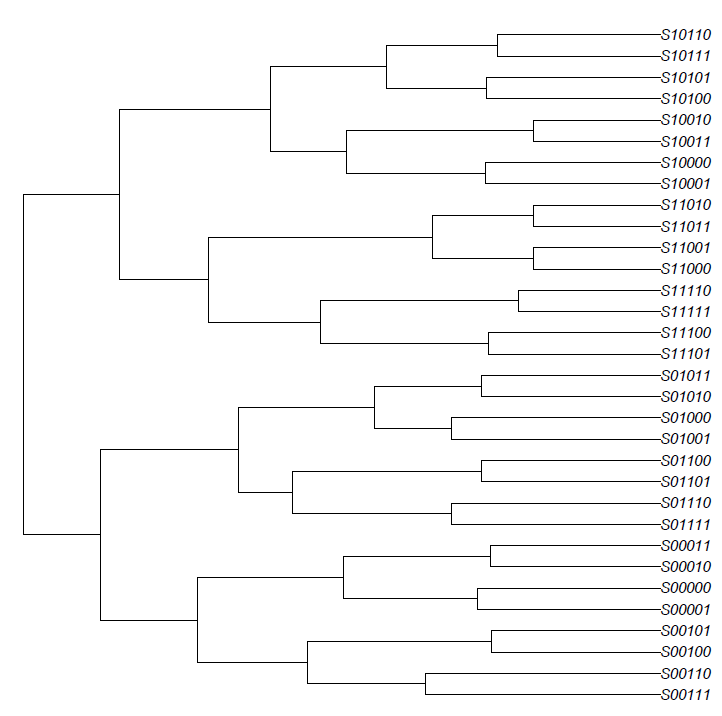


Figure S2: Phylogenetic tree reconstructed based on multiple alignment by OMMA of simulated genomes. The genome names refer to the path of how they diverged from the origin in simulation (See Method section for details).


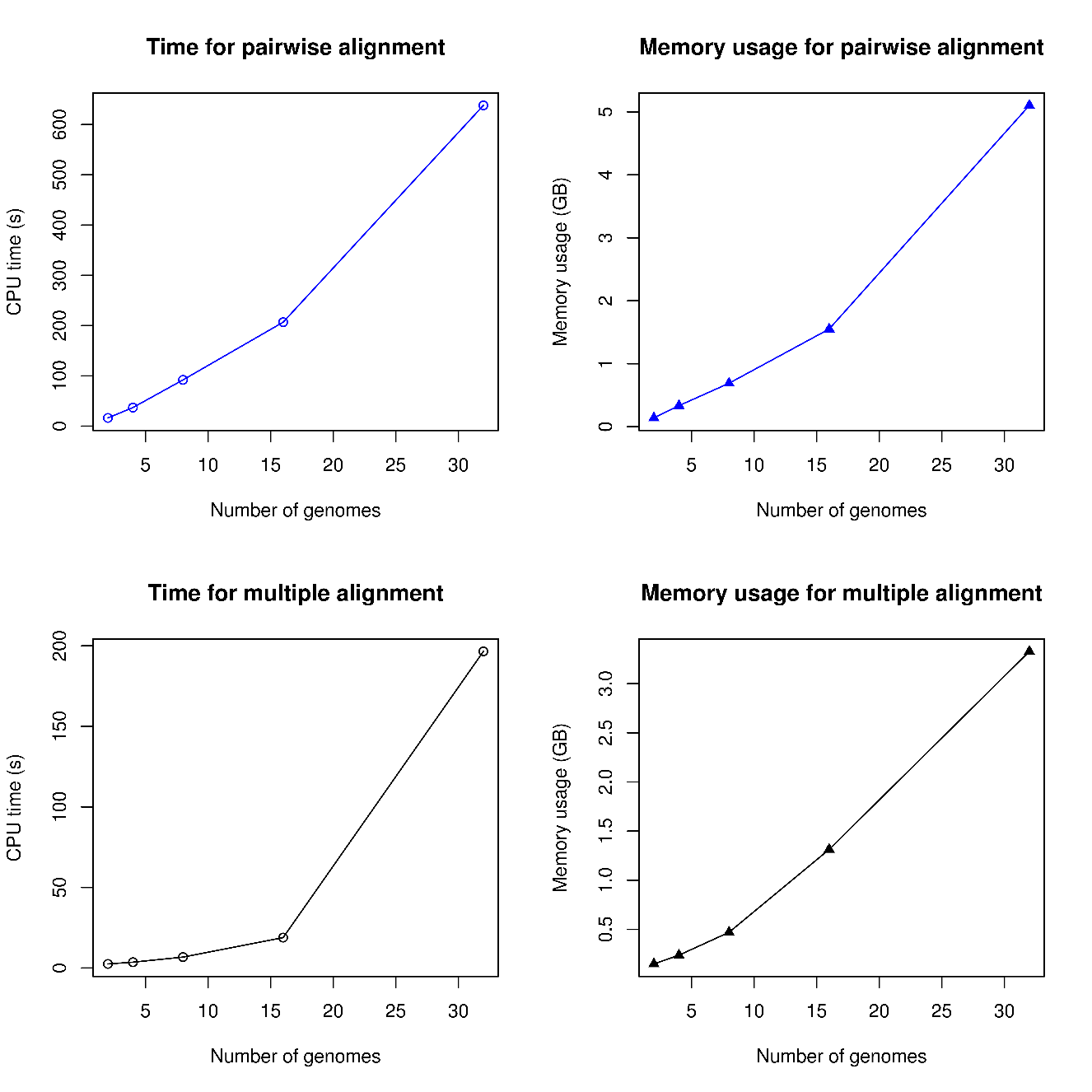


Figure S3: CPU time and memory usage for pairwise alignment and multiple alignment of different number of *A. Baumannii* genomes


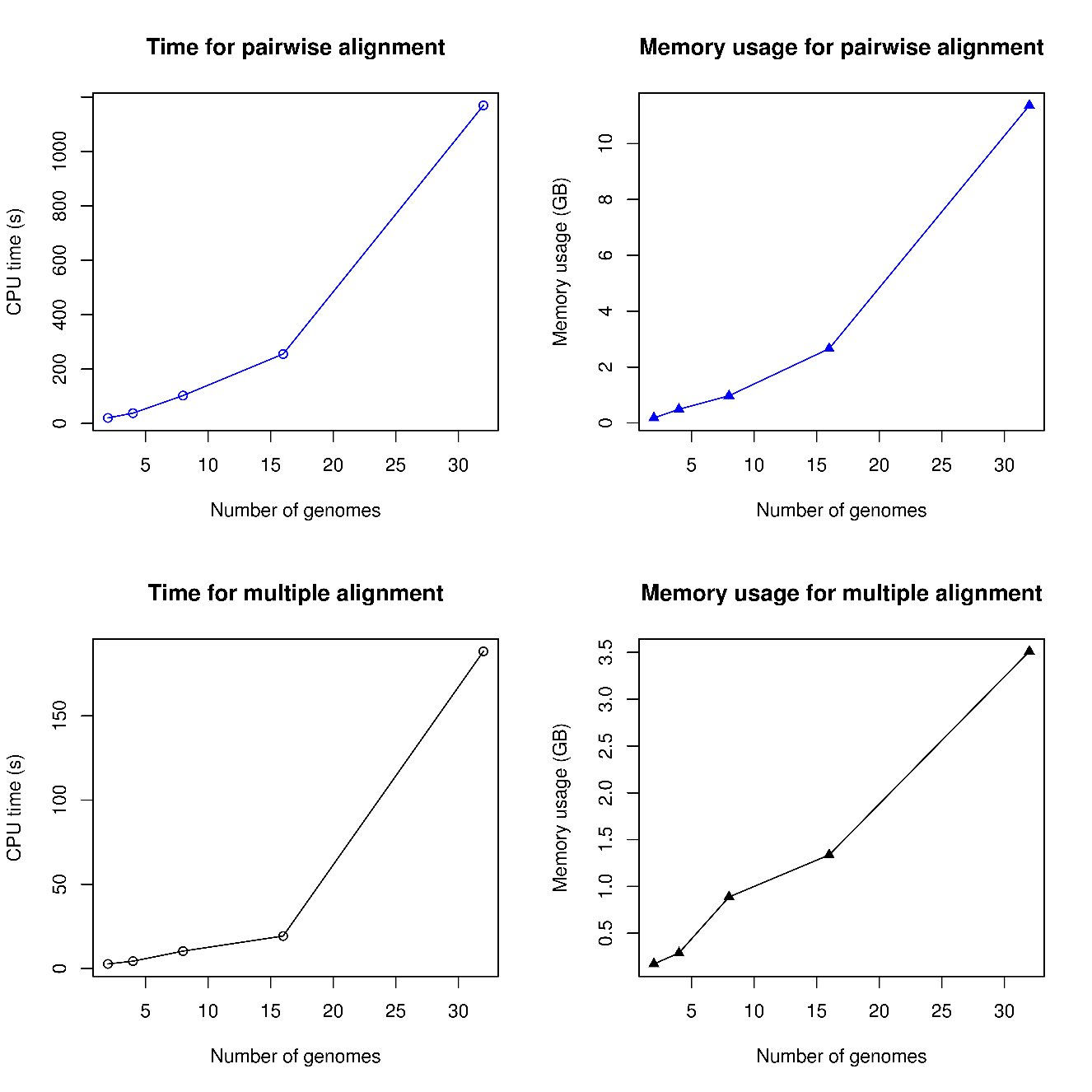


Figure S4: CPU time and memory usage for pairwise alignment and multiple alignment of different number of *E. coli* genomes


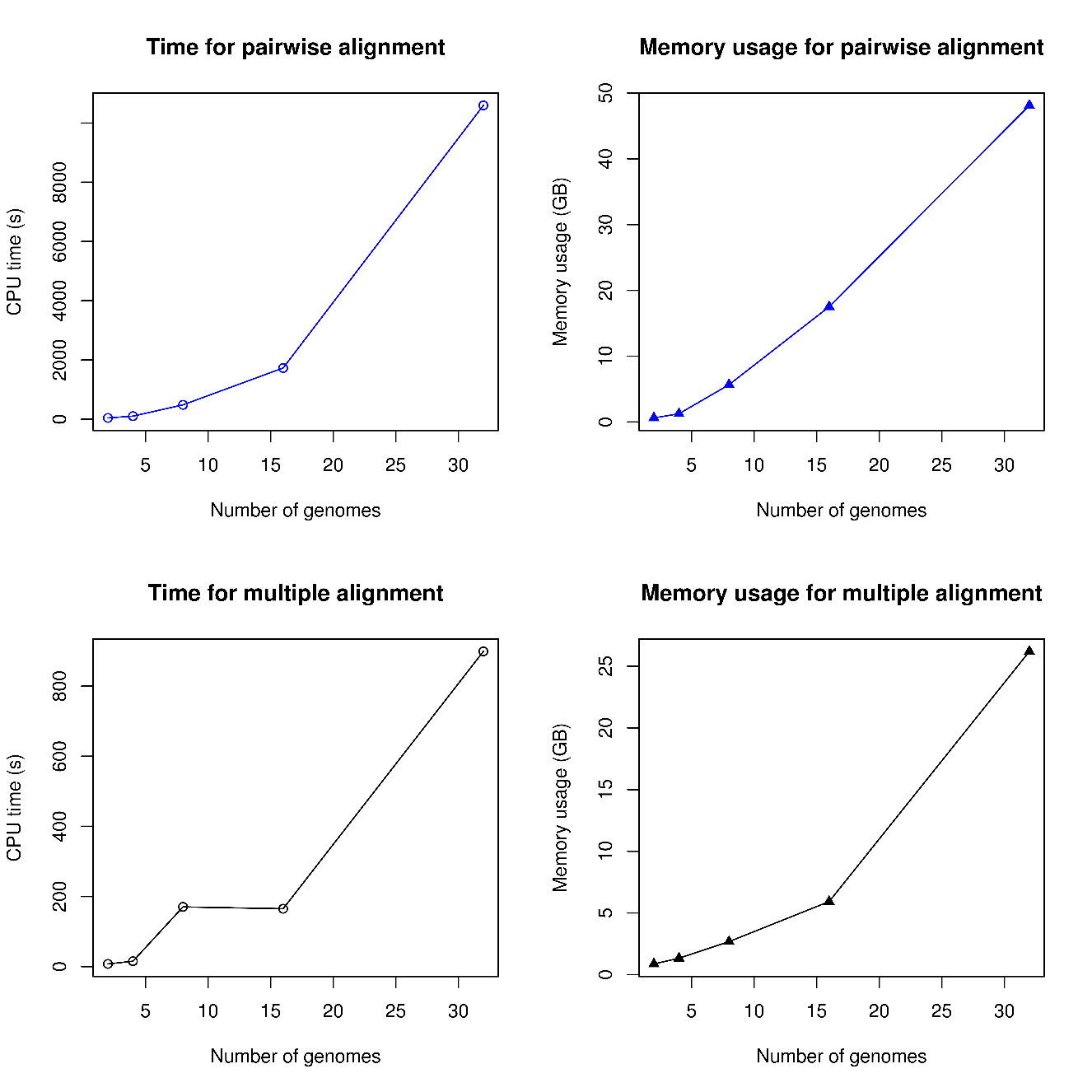


Figure S5: CPU time and memory usage for pairwise alignment and multiple alignment of different number of *S. cerevisiae* genomes


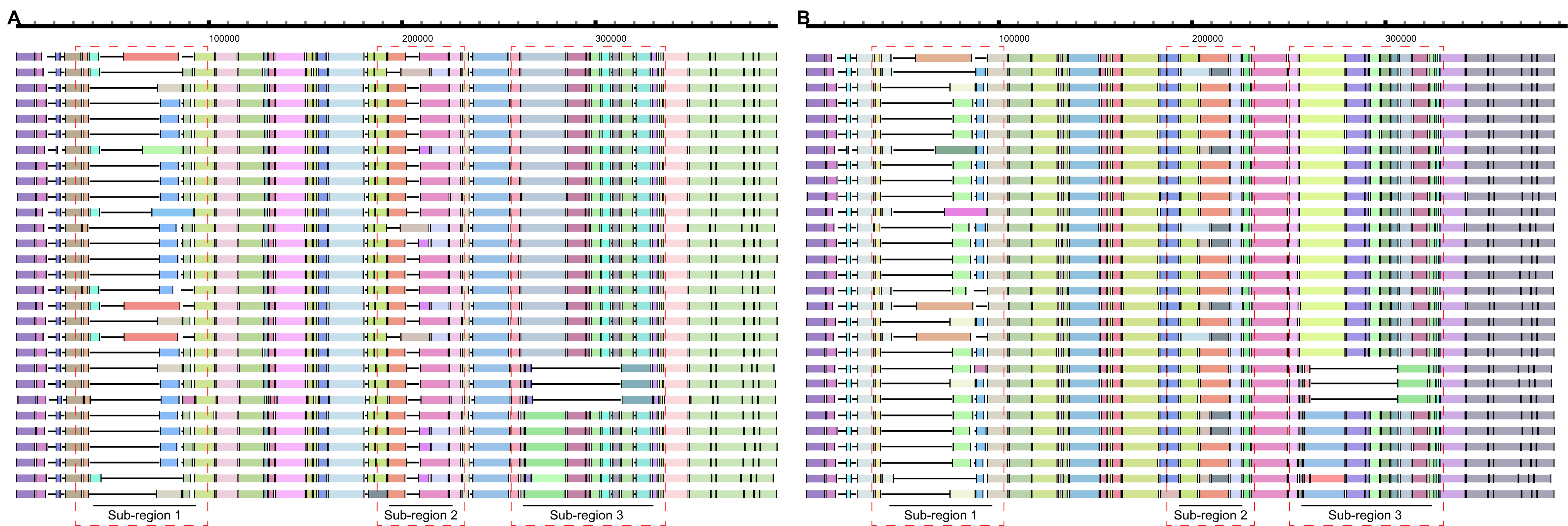


Figure S6: Multiple alignment by OMMA of the contigs with (A) and without (B) hg38 of Figure 2 using the same parameters.


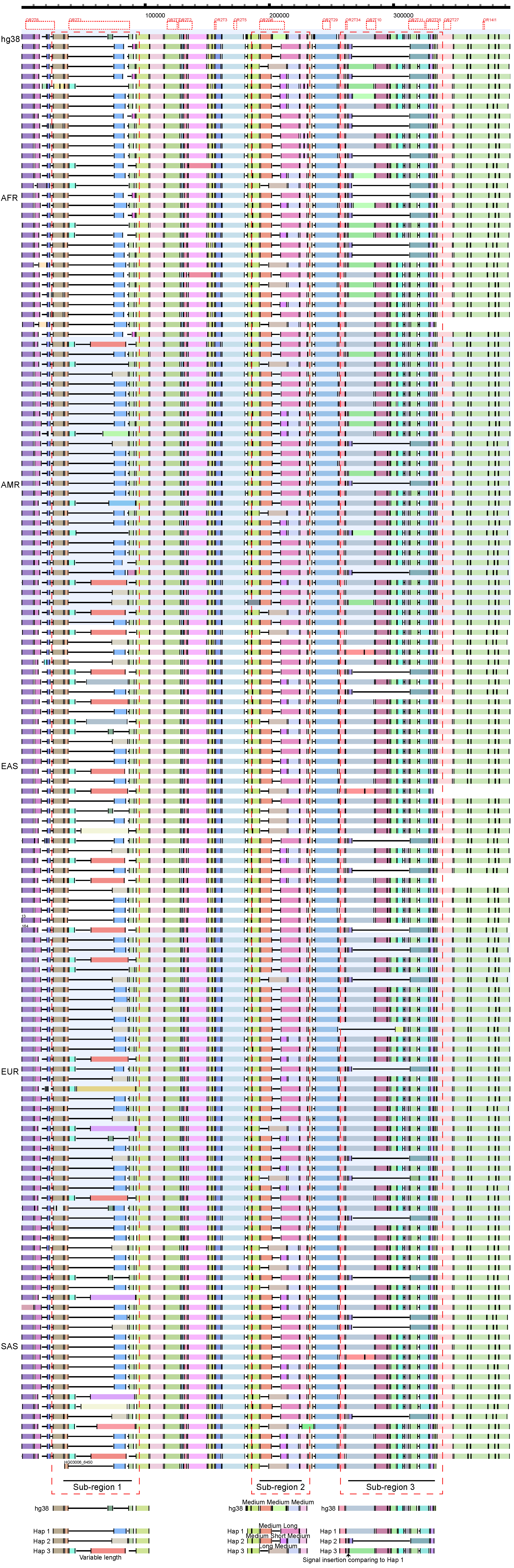


Figure S7: Multiple alignment by OMMA of the contigs from all populations at the olfactory receptor region (1q44).


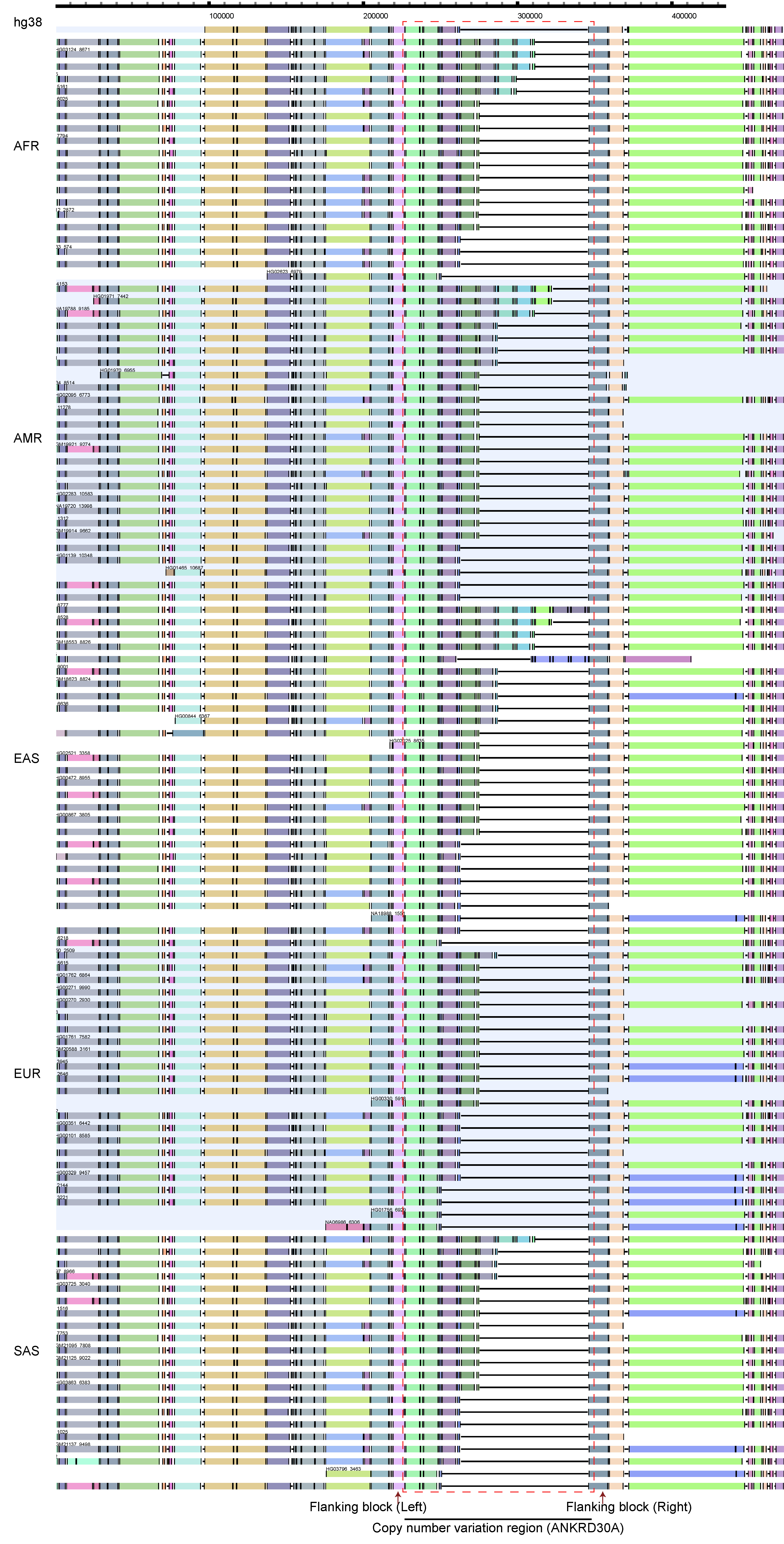


Figure S8: Multiple alignment of contigs by OMMA from all populations at gene *ANKRD30A*. The European contigs possessed fewer copies than the African, American, and East Asian contigs.


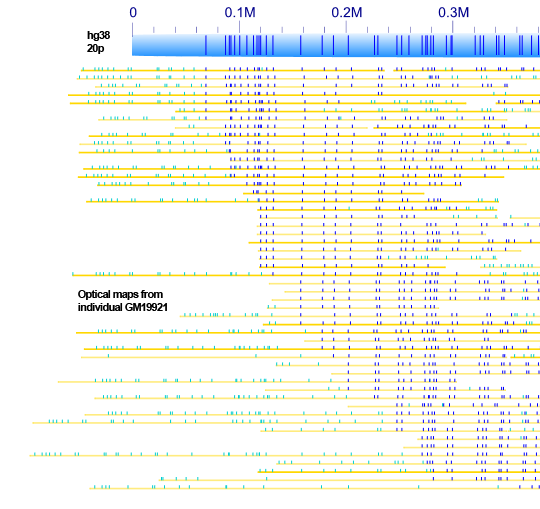


Figure S9: Alignment of optical maps from individual GM19921 on chromosome 20 using IrysView. Optcal maps could be observed that extend beyond the hg38 chromosome 20p, but it is hard to distinguish and characterize the labeling patterns when multiple haplotypes exist. The consensus pattern of these optical maps was multiply aligned with consensus patterns from other individuals in Figure 5.


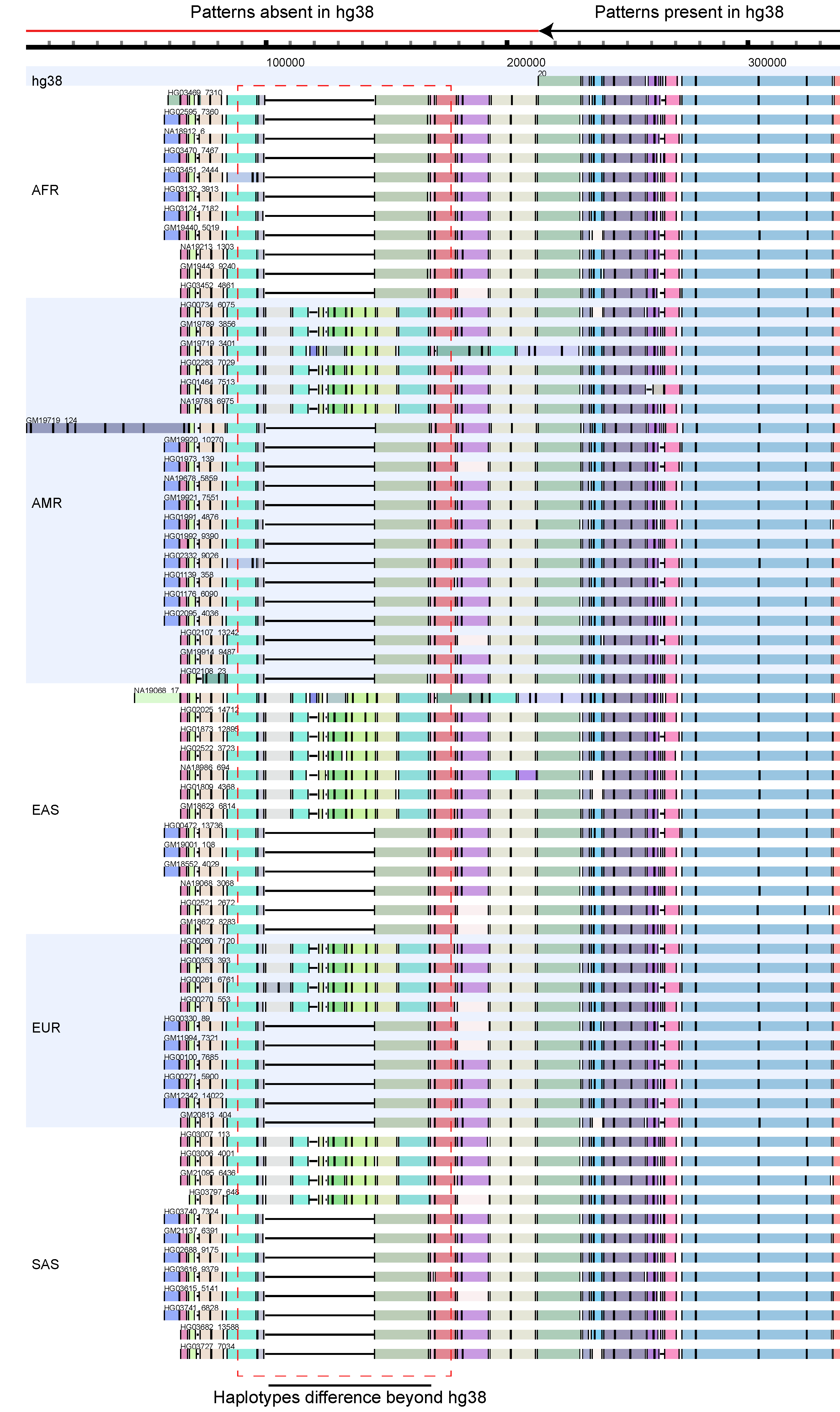


Figure S10: Multiple alignment of the contigs by OMMA from all populations at the subtelomeric region of chromosome 20p. The two major haplotypes were visualized beyond hg38. The African contigs only contained the shorter form of the haplotype, while the other ethnic groups contained both the longer and shorter forms.


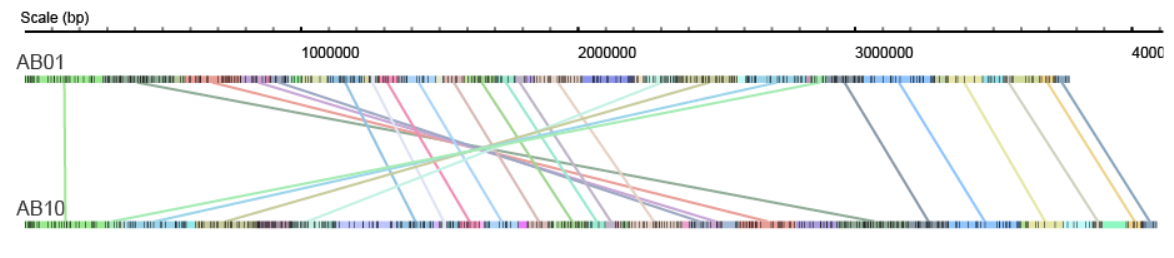


Figure S11. Example of genome rearrangement. The genome structure of AB10 is different from that of AB1.


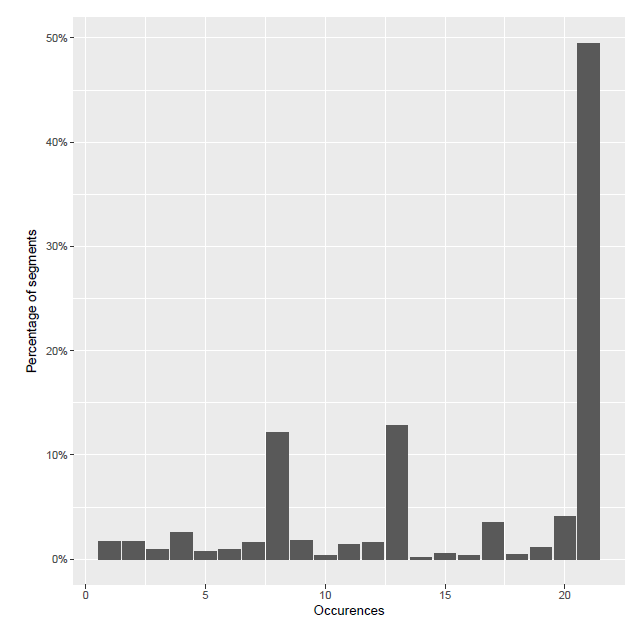


Figure S12: Occurrence of segments. A segment has *x* occurrences if the block it belongs to contains segments from *x* queries. A segment that occurs once implies it is unique to its parent genome and does not match with any segment from other genomes. In contrast, with 21 occurrences, the segment has counterparts from all 21 genomes in the multiple alignment.


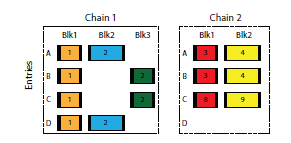


Figure S13: An example showing the connection of two chains of blocks. The relationship of four sets of entries from query A, B, C, and D represented matching, direct matching, rearrangement, and empty relationships, respectively.


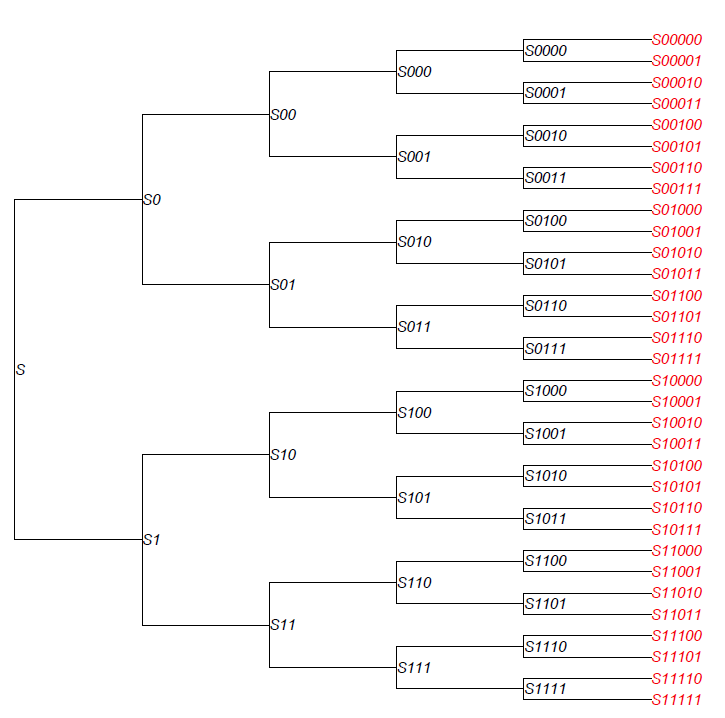


Figure S14: Data simulation for phylogenetic tree assessment. A source genome "S" was taken as the ancestor genome. At each generation, two different children genomes were simulated by introducing random mutations into the parent genome, which had their names denoted as their parent genome name with the addition of suffix “0” or “1”. The children genomes at the last generation (Red) were used in the analysis.
