# Supplementary material for: OMMA enables population-scale analysis of complex genomic features and phylogenomic relationships from nanochannel-based optical maps
Source: Gigascience. 2019 Jul 9;8(7):giz079. doi: 10.1093/gigascience/giz079 (PMC6615982; doi:10.1093/gigascience/giz079)
Supplement: giz079_Response_to_Reviewer_Comments_Original_Submission [file giz079_response_to_reviewer_comments_original_submission.pdf]

Author's Response To Reviewer Comments

Close

Reviewer 1
Major
1. Let's consider multiple DNA sequence alignment. The only "true" method of solving multiple sequence alignment is to extend smith-watermen pairwise alignment to multiple sequence alignment. Following this paradigm provides a O(n^k)-time and -space algorithm for finding the multiple alignment between k sequences of length <= n. This method has been repeatedly showed not to be efficient in practice. Thus, there are a number of heuristics for solving multiple sequence alignment, majority find (n choose k) pairwise alignments either exactly or using some heuristic.
Multiple optical map alignment is no different. OMMA is a heuristic and does not guarantee to find an optimal multiple alignment. Thus, given this fact and the fact that there are A LOT of methods for finding an alignment between a pair optical maps (Twin, SOMA, Valouev, OMBlast, etc..) it makes no sense not to compare against these methods. Hence, the authors have to compare against these existing methods (by using them to find (n choose k) alignments).

Response: Thanks for the detailed elaboration. We appreciate the fact that there are many existing methods for finding optimal pairwise alignment between a pair of optical maps. However, the main focus of this manuscript is on our new OMMA module for multiple alignment and its applications, rather than on which pairwise alignment method is best suited as input for multiple alignment. Adding a comparison of pairwise alignment methods would very much divert our intended focus. We should also emphasize that our multiple alignment module accepts pairwise alignment outputs from all these tools.

2. CPU-usage, disk-usage, and running time need to be compared in order to assess how efficient (or inefficient) OMMA is. The authors need to demonstrate how the running time and needed computing resources scale with respect to the size of the dataset (i.e., varying the number of optical maps, and the genome size).

Response: Thanks for the suggestion. We have now added a new section describing computational resource requirement of OMMA in the Supplementary text.

3. How was the simulated data generated? An existing 3rd-party method should be used for simulation, i.e., OMSim.

Response: The simulated genome was generated using pIRS program (Bioinformatics 28:1533-35 2012), where random mutations were added to the genome sequence. OMSim, however, only simulates optical maps data instead. We have changed the text to make this point clearer. (Line 392-402)

4. The performance of OMMA needs to be compared as the frequency of the errors increases. For example, OMSim allows a number of the input parameters that control the error rate to be varied. The authors should vary the error rate and give the precision and recall (AUC).

Response: As the assembly process already handled most of the errors in raw optical maps, varying the error rates would in fact affect the assembly quality instead of the OMMA performance. Hence, we only used error-free simulated genomes for the performance analysis.

5. There are standard statistics for measuring the optimality of an alignment. For example, the S-score. Thus, the S-score can be used to compare the multiple alignments produced by OMMA with those of the methods described in (1).

Response: As explained in our response to (1), the methods mentioned are on pairwise alignment while

our OMMA module makes use of the pairwise alignment results to perform multiple alignment.

Minor

1. There a number of alignment methods that are missing, i.e., Twin (Muggli et al.), SOMA, etc.

Response: Thank you for reminding us, we have now included these alignment methods. (Line 45)

2. Recently there exists methods to error correct the optical mapping data (such as Comet (GigaScience 2018). I would suggest the authors should try running the error correction method prior to running OMMA since it may boost their results.

Response: Thanks for the suggestions. It is interesting to see how error correction affects the assembly quality. However, the analysis is beyond the scope of this manuscript. A discussion is added in the text. (Line 397-401)

3. For the simulated results, the authors compare the multiple alignment produced by OMMA with that of the multiple sequence alignment; yet, the authors do not describe how the multiple sequence alignment was produced.

Response: We previously described the multiple sequence alignment under the section "Assessment of accuracy of OMMA". We have modified the respective sentences for clarity.
"In this study, the multiple sequence alignment was performed using Mugsy v1r2.3 [15] which used results from MUMmer 3.20 [16] under the default parameters." (Line 367-369)

4. Similar to (4), I would suggest comparing the phylogenetics of produced using OMMA with the phylogenetics produced using the sequence data; or describe why this cannot be accomplished.

Response: The comparison of phylogenetic tree reconstructed based on MLST (based on sequence data) and OMMA (based on optical map data) has been described in Figure 5.

Reviewer 2
Major
1. I would have liked to see some discussion about how different errors in the optical mapping process such as missed or inserted nicking site errors affect the multiple alignment of the maps. Also, how does your method addresses these challenges caused due to errors?

Response: The main purpose of OMMA is to perform multiple alignment on optical mapping assemblies rather than raw optical maps. The errors in raw data is being handled during the de novo assembly procedure using Bionano Genomics' in-house assembly tool. This is the main reason why we employed simulated error-free assembly for multiple alignment. We have added a subsection "Generation of simulated genomes" to improve the clarity on how simulated genomes were generated. (Line 392-402)

2. What are the computational requirements of the method? How much time and memory was required for your experiments and how does the runtime increase as you try to align more genomes? Some discussion about the computational complexity of the method will give an idea regarding this.

Response: Thank you for the suggestion. We have now added a new section in the Supplementary text describing computational resource requirement for OMMA.

Minor
1. Page 3 line 70-71: Here error-free simulated maps were used. Again some discussion if not experiments are useful to stress how errors can affect the method.

Response: The raw data quality is handled during the assembly process. This is the main reason for employing simulated error-free assembly for multiple alignment purpose. We have modified the text and

discussed the effects of minor assembly error in the subsection “Generation of simulated genomes”. (Line 392-402)

2. Page 4 line 80: "This reconstructed tree has the same topology as the simulated tree". This statement is weak. Some quantitive measure to stress this point will be helpful.

Response: We have re-run the tree reconstruction on 100 random sets of simulated genomes. They are now described in the performance analysis section.

3. Page 4 line 90-91: You mentioned before that multiple alignment is a reference-free method to study regions of variation, but here you seem to be using the hg38. What happens if you don't use it? Also, please be specific when you mean by "better biological interpretation".

Response: The inclusion of hg38 allowed us to annotate the variations. We have rewritten the sentence as:
“We used OMMA to perform multiple alignment of these contigs with part of the reference genome hg38 used as one of the queries to obtain annotations related to the variations.“ (Line 96-99)

As hg38 is only one of all the queries, it does not affect the multiple alignment result much. Please see the multiple alignment in Figure 2 with and without hg38 using the same parameters in Figure S2.

4. Page 4 line 96: Better in terms of what?

Response: We actually wanted to emphasize that pairwise alignment only provides presence and absence of variation but multiple alignment summarizes all the variation types. We have removed the word “better” and rewritten the sentence as:
“Multiple alignment, however, provides a summary of all haplotypes present in the region.” (Line 103)

5. Page 5 line 124: How do you choose the two MA-blocks that flank the CNV region?

Response: They were chosen because they did not show similar patterns as the repeat units. Hence, they corresponded to the boundary of the CNV region.

6. Page 6 line 144-145: In this section, do you observe similar results when you use sequencing data and do multiple alignment? If no, what type of regions you would miss which would be captured by optical mapping data? It would help highlight the use of optical mapping and your method do to such an analysis over the sequencing data.

Response: The major type of regions missing are those that could not be easily assembled. We have edited the following in the discussion part.
“The assembled optical mapping contigs are usually much longer than sequence assemblies that break into pieces at short repetitive regions.” (Line 206)

Close
