# Supplementary material for: OMMA enables population-scale analysis of complex genomic features and phylogenomic relationships from nanochannel-based optical maps
Source: Gigascience. 2019 Jul 9;8(7):giz079. doi: 10.1093/gigascience/giz079 (PMC6615982; doi:10.1093/gigascience/giz079)
Supplement: giz079_Response_to_Reviewer_Comments_Revision_1 [file giz079_response_to_reviewer_comments_revision_1.pdf]

Author's Response To Reviewer Comments

Close

EDITORIAL

1. Note that the reviewer highlights that the assembly process will not be able to handle most of the errors, and they insist that you need to vary the error rate (since this technology is new and the error rate varies widely), assemble, and then run your method. Note that both reviewers independently requested this. Thus, it is important and requires further experimentation. You need to add more information in terms of how the method performs as the error rate increases. And we agree with the reviewer in that methods should be included in the main manuscript, not in the supplemental files where it can eventually get lost.

Response: We now have fully addressed the concerns raised by the reviewer, and our point-by-point responses are listed below.

2. In addition, please register any new software application in the SciCrunch.org database to receive a RRID (Research Resource Identification Initiative ID) number, and include this in your manuscript. This will facilitate tracking, reproducibility and re-use of your tool.

Response: The resource has been submitted to SciCrunch.org with the RRID SCR_017143.

REVIEWER 1

Response: Thanks for the detailed elaboration. We appreciate the fact that there are many existing methods for finding optimal pairwise alignment between a pair of optical maps. However, the main focus of this manuscript is on our new OMMA module for multiple alignment and its applications, rather than on which pairwise alignment method is best suited as input for multiple alignment. Adding a comparison of pairwise alignment methods would very much divert our intended focus. We should also emphasize that our multiple alignment module accepts pairwise alignment outputs from all these tools.
Reviewer Response: That is fine. Yet, you should still cite this work to put this manuscript into context. The point is, a lot of multiple alignment software methods (for sequence data) is preforming n choose 2 pairwise alignments.

Response: We have made citations to the suggested methods.

Response: Thanks for the suggestion. We have now added a new section describing computational resource requirement of OMMA in the Supplementary text.
Reviewer Response: Given that this was a request by both reviewers, it should go in the main document. This is very important. Please make space for it in the main document.

Response: We have moved the section to the main document, under the section computational resources analysis (Lines 90 to 96.

Response: As the assembly process already handled most of the errors in raw optical maps, varying the error rates would in fact affect the assembly quality instead of the OMMA performance. Hence, we only used error-free simulated genomes for the performance analysis.
Reviewer Response. The assembly process will not be able to handle most of the errors. I still insist that the authors' need to vary the error rate (since this technology is new and the error rate varies widely), assemble, and then run their method. Note that both reviewers independently requested this. Thus, it is

important and requires further experimentation.

Response: We have added the performance analysis with various errors introduced at different rates. The new results are described in the performance analysis section (Lines 75 to 78; 391 to 398) and in the supplementary figure S14. Briefly, we have shown that the precision and recall of OMMA remains high until the error rates reach a level so high that is usually not seen in typical optical mapping dataset.

Close
