# Supplementary material for: OMMA enables population-scale analysis of complex genomic features and phylogenomic relationships from nanochannel-based optical maps
Source: Gigascience. 2019 Jul 9;8(7):giz079. doi: 10.1093/gigascience/giz079 (PMC6615982; doi:10.1093/gigascience/giz079)
Supplement: giz079_Reviewer_1_Report_Original_Submission — Christina Boucher -- 9/1/2018 Reviewed [file giz079_reviewer_1_report_original_submission.pdf]

Reviewer Report

Title: OMMA enables population-scale analysis of complex genomic features and phylogenomic relationships from nanochannel-based optical maps

Version: Original Submission **Date:** 9/1/2018

Reviewer name: Christina Boucher

Reviewer Comments to Author:

Here, the authors develop a method for finding a multiple alignment for population-level analysis of optical mapping data. They refer to their method as optical mapping by multiple alignment (OMMA). Although there are a number of methods that find pairwise alignments between species, to the best of my knowledge, there does not exist a method for finding a mulitple alignment between optical maps. Since the problem is novel and has not been yet solved, it is worthy of further investigation. The authors describe the OMMA algorithm and demonstrate its effectiveness using both simulated and experimental data.

My overall assessment of this paper is that although the problem and approach are interesting, the experimental section needs major revision before it can be accepted for publication.

Below is a list of my major and minor concerns of the paper.

Major:

1. Let's consider multiple DNA sequence alignment. The only "true" method of solving multiple sequence alignment is to extend smith-watermen pairwise alignment to multiple sequence alignment. Following this paradigm provides a O(n^k)-time and -space algorithm for finding the multiple alignment between k sequences of length <= n. This method has been repeatedly showed not to be efficient in practice. Thus, there are a number of heuristics for solving multiple sequence alignment, majority find (n choose k) pairwise alignments either exactly or using some heuristic.

Multiple optical map alignment is no different. OMMA is a heuristic and does not guarantee to find an optimal multiple alignment. Thus, given this fact and the fact that there are A LOT of methods for finding an alignment between a pair optical maps (Twin, SOMA, Valouev, OMBlast, etc..) it makes no sense not to compare against these methods. Hence, the authors have to compare against these existing methods (by using them to find (n choose k) alignments). CPU-usage, disk-usage, and running time need to be compared in order to assess how efficient (or inefficient) OMMA is.

2. The authors needs to demonstrate how the running time and needed computing resources scale with respect to the size of the dataset (i.e., varying the number of optical maps, and the genome size).

3. How was the simulated data generated? An existing 3rd-party method should be used for simulation, i.e., OMSim.

4. The performance of OMMA needs to be compared as the frequency of the errors increases. For example, OMSim allows a number of the input parameters that control the error rate to be

varied. The authors should vary the error rate and give the precision and recall (AUC).
5. There are standard statistics for measuring the optimality of an alignment. For example, the S-score. Thus, the S-score can be used to compare the multiple alignments produced by OMMA with those of the methods described in (1).
Minor:
1. There a number of alignment methods that are missing, i.e., Twin (Muggli et al.), SOMA, etc.
2. Recently there exists methods to error correct the optical mapping data (such as Comet (GigaScience 2018). I would suggest the authors should try running the error correction method prior to running OMMA since it may boost their results.
3. For the simulated results, the authors compare the multiple alignment produced by OMMA with that of the multiple sequence alignment; yet, the authors do not describe how the multiple sequence alignment was produced.
4. Similar to (4), I would suggest comparing the phylogenetics of produced using OMMA with the phylogenetics produced using the sequence data; or describe why thise cannot be accomplished.

Level of Interest

Please indicate how interesting you found the manuscript: Choose an item.

Quality of Written English

Please indicate the quality of language in the manuscript: Choose an item.

Declaration of Competing Interests

Please complete a declaration of competing interests, considering the following questions:

- Have you in the past five years received reimbursements, fees, funding, or salary from an organisation that may in any way gain or lose financially from the publication of this manuscript, either now or in the future?
- Do you hold any stocks or shares in an organisation that may in any way gain or lose financially from the publication of this manuscript, either now or in the future?
- Do you hold or are you currently applying for any patents relating to the content of the manuscript?
- Have you received reimbursements, fees, funding, or salary from an organization that holds or has applied for patents relating to the content of the manuscript?
- Do you have any other financial competing interests?
- Do you have any non-financial competing interests in relation to this paper?

If you can answer no to all of the above, write 'I declare that I have no competing interests' below. If your reply is yes to any, please give details below.

I declare that I have no competing interests.

I agree to the open peer review policy of the journal. I understand that my name will be included on my report to the authors and, if the manuscript is accepted for publication, my named report including any attachments I upload will be posted on the website along with the authors' responses. I agree for my report to be made available under an Open Access Creative Commons CC-BY license (http://creativecommons.org/licenses/by/4.0/). I understand that any comments which I do not wish to be included in my named report can be included as confidential comments to the editors, which will not be published.

I agree to the open peer review policy of the journal

To further support our reviewers, we have joined with Publons, where you can gain additional credit to further highlight your hard work (see: https://publons.com/journal/530/gigascience). On publication of this paper, your review will be automatically added to Publons, you can then choose whether or not to claim your Publons credit. I understand this statement.

Yes Choose an item.
