# Supplementary material for: OMMA enables population-scale analysis of complex genomic features and phylogenomic relationships from nanochannel-based optical maps
Source: Gigascience. 2019 Jul 9;8(7):giz079. doi: 10.1093/gigascience/giz079 (PMC6615982; doi:10.1093/gigascience/giz079)
Supplement: giz079_Reviewer_1_Report_Revision_1 — Christina Boucher -- 1/28/2019 Reviewed [file giz079_reviewer_1_report_revision_1.pdf]

Reviewer Report

Title: OMMA enables population-scale analysis of complex genomic features and phylogenomic relationships from nanochannel-based optical maps

Version: Revision 1 **Date:** 1/28/2019

Reviewer name: Christina Boucher

Reviewer Comments to Author:

Reviewer 1
Response: Thanks for the detailed elaboration. We appreciate the fact that there are many existing methods for finding optimal pairwise alignment between a pair of optical maps. However, the main focus of this manuscript is on our new OMMA module for multiple alignment and its applications, rather than on which pairwise alignment method is best suited as input for multiple alignment. Adding a comparison of pairwise alignment methods would very much divert our intended focus. We should also emphasize that our multiple alignment module accepts pairwise alignment outputs from all these tools.
Reviewer Response: That is fine. Yet, you should still cite this work to put this manuscript into context. The point is, a lot of multiple alignment software methods (for sequence data) is preforming n choose 2 pairwise alignments.
Response: Thanks for the suggestion. We have now added a new section describing computational resource requirement of OMMA in the Supplementary text.
Reviewer Response: Given that this was a request by both reviewers, it should go in the main document. This is very important. Please make space for it in the main document.
Response: The simulated genome was generated using pIRS program (Bioinformatics 28:1533-35 2012), where random mutations were added to the genome sequence. OMSim, however, only simulates optical maps data instead. We have changed the text to make this point clearer. (Line 392-402)
Reviewer Response: Thank you. That clarifies it.
Response: As the assembly process already handled most of the errors in raw optical maps, varying the error rates would in fact affect the assembly quality instead of the OMMA performance. Hence, we only used error-free simulated genomes for the performance analysis.
Reviewer Response. The assembly process will not be able to handle most of the errors. I still insist that the authors' need to vary the error rate (since this technology is new and the error rate varies widely), assemble, and then run their method. Note that both reviewers indenpendently requested this. Thus, it is important and requires further experimentation.
Response: As explained in our response to (1), the methods mentioned are on pairwise alignment while our OMMA module makes use of the pairwise alignment

results to perform multiple alignment.
Reviewer Response: Thank you.
- My minor revisions have been taken care of. Thank you.

Level of Interest

Please indicate how interesting you found the manuscript: Choose an item.

Quality of Written English

Please indicate the quality of language in the manuscript: Choose an item.

Declaration of Competing Interests

Please complete a declaration of competing interests, considering the following questions:

- Have you in the past five years received reimbursements, fees, funding, or salary from an organisation that may in any way gain or lose financially from the publication of this manuscript, either now or in the future?
- Do you hold any stocks or shares in an organisation that may in any way gain or lose financially from the publication of this manuscript, either now or in the future?
- Do you hold or are you currently applying for any patents relating to the content of the manuscript?
- Have you received reimbursements, fees, funding, or salary from an organization that holds or has applied for patents relating to the content of the manuscript?
- Do you have any other financial competing interests?
- Do you have any non-financial competing interests in relation to this paper?

If you can answer no to all of the above, write 'I declare that I have no competing interests' below. If your reply is yes to any, please give details below.

I declare that I have no competing interests.

I agree to the open peer review policy of the journal. I understand that my name will be included on my report to the authors and, if the manuscript is accepted for publication, my named report including any attachments I upload will be posted on the website along with the authors' responses. I agree for my report to be made available under an Open Access Creative Commons CC-BY license (http://creativecommons.org/licenses/by/4.0/). I understand that any comments which I do not wish to be included in my named report can be included as confidential comments to the editors, which will not be published.

I agree to the open peer review policy of the journal

To further support our reviewers, we have joined with Publons, where you can gain additional credit to further highlight your hard work (see: https://publons.com/journal/530/gigascience). On publication of this paper, your review will be automatically added to Publons, you can then choose whether or not to claim your Publons credit. I understand this statement.

Yes Choose an item.
