# Supplementary material for: OMMA enables population-scale analysis of complex genomic features and phylogenomic relationships from nanochannel-based optical maps
Source: Gigascience. 2019 Jul 9;8(7):giz079. doi: 10.1093/gigascience/giz079 (PMC6615982; doi:10.1093/gigascience/giz079)
Supplement: giz079_Reviewer_2_Report_Original_Submission — Jay Ghurye -- 10/11/2018 Reviewed [file giz079_reviewer_2_report_original_submission.pdf]

Reviewer Report

Title: OMMA enables population-scale analysis of complex genomic features and phylogenomic relationships from nanochannel-based optical maps

Version: Original Submission **Date:** 10/11/2018

Reviewer name: Jay Ghurye

Reviewer Comments to Author:

In this manuscript, the authors describe a method OMMA to better understand complex genomic features. They showed the application of their method on Acinetobacter baumannii genome and on 154 human samples. Overall, the paper is written nicely. Here are my comments
Methods:
I would have liked to see some discussion about how different errors in the optical mapping process such as missed or inserted nicking site errors affect the multiple alignment of the maps. Also, how does your method addresses these challenges caused due to errors?
What are the computational requirements of the method? How much time and memory was required for your experiments and how does the runtime increase as you try to align more genomes? Some discussion about the computational complexity of the method will give an idea regarding this.
Results:
Page 3 line 70-71: Here error-free simulated maps were used. Again some discussion if not experiments are useful to stress how errors can affect the method.
Page 4 line 80: "This reconstructed tree has the same topology as the simulated tree". This statement is weak. Some quantitive measure to stress this point will be helpful.
Page 4 line 90-91: You mentioned before that multiple alignment is a reference-free method to study regions of variation, but here you seem to be using the hg38. What happens if you don't use it? Also, please be specific when you mean by "better biological interpretation".
Page 4 line 96: Better in terms of what?
Page 5 line 124: How do you choose the two MA-blocks that flank the CNV region?
Page 6 line 144-145: In this section, do you observe similar results when you use sequencing data and do multiple alignment? If no, what type of regions you would miss which would be captured by optical mapping data? It would help highlight the use of optical mapping and your method do to such an analysis over the sequencing data.

Level of Interest

Please indicate how interesting you found the manuscript: Choose an item.

Quality of Written English

Please indicate the quality of language in the manuscript: Choose an item.

Declaration of Competing Interests

Please complete a declaration of competing interests, considering the following questions:

- Have you in the past five years received reimbursements, fees, funding, or salary from an organisation that may in any way gain or lose financially from the publication of this manuscript, either now or in the future?
- Do you hold any stocks or shares in an organisation that may in any way gain or lose financially from the publication of this manuscript, either now or in the future?
- Do you hold or are you currently applying for any patents relating to the content of the manuscript?
- Have you received reimbursements, fees, funding, or salary from an organization that holds or has applied for patents relating to the content of the manuscript?
- Do you have any other financial competing interests?
- Do you have any non-financial competing interests in relation to this paper?

If you can answer no to all of the above, write 'I declare that I have no competing interests' below. If your reply is yes to any, please give details below.

I declare that I have no competing interests

I agree to the open peer review policy of the journal. I understand that my name will be included on my report to the authors and, if the manuscript is accepted for publication, my named report including any attachments I upload will be posted on the website along with the authors' responses. I agree for my report to be made available under an Open Access Creative Commons CC-BY license (http://creativecommons.org/licenses/by/4.0/). I understand that any comments which I do not wish to be included in my named report can be included as confidential comments to the editors, which will not be published.

I agree to the open peer review policy of the journal

To further support our reviewers, we have joined with Publons, where you can gain additional credit to further highlight your hard work (see: https://publons.com/journal/530/gigascience). On publication of this paper, your review will be automatically added to Publons, you can then choose whether or not to claim your Publons credit. I understand this statement.

Yes Choose an item.
